# Bioactive flavonoids from *Anacardium occidentale* as promising natural inhibitors of *Cryptococcus neoformans*: a computational perspective on secondary metabolites against critical fungal pathogens

**DOI:** 10.1007/s00203-026-04814-9

**Published:** 2026-03-03

**Authors:** Marcus Vinícius Ferreira da Silva, Jacilene Silva, Victor Moreira de Oliveira, Matheus Nunes da Rocha, Selene Maia de Morais, Emmanuel Silva Marinho

**Affiliations:** 1 Postgraduate Program in Veterinary Sciences, State University of Ceará, Fortaleza, Ceará Brazil; 2 Department of Biological Chemistry, Regional University of Cariri, Crato, Ceará Brazil; 3 Postgraduate Program in Biotechnology, State University of Ceará, Fortaleza, Ceará Brazil; 4 Postgraduate Program in Natural Sciences, State University of Ceará, Fortaleza, Brazil; 5 Science and Technology Center–Chemistry Course, State University of Ceará, Fortaleza, Brazil

**Keywords:** *Anacardium occidentale*, *Cryptococcus neoformans*, Molecular docking, Molecular dynamics, Phytochemicals, Pharmacokinetics

## Abstract

The investigation of bioactive phytochemicals from *Anacardium occidentale* (cashew) nut has attracted increasing interest, particularly due to their potential antifungal properties against *Cryptococcus neoformans*. In this study, molecular docking analysis were employed to assess the interaction strength of selected compounds with crucial enzymatic targets, including farnesyltransferase (CnFTase), beta-carbonic anhydrase (β-CA), and adenylosuccinate synthetase (AdSS). Molecular dynamics simulation was performed using the iMODS server, in order to check the stability as well as mobility in the receptor-ligand complexes following molecular docking. To evaluate the pharmacokinetic behavior, a multiparametric optimization (MPO) approach was applied, considering parameters such as membrane permeability (PAMPA), metabolic processing via cytochrome P450 (CYP450) enzymes, and clearance rates (Cl_int, u_, Cl_Micro_, Cl_Hepa_). The degree to which the ligands resemble drugs was evaluated using drug-likeness scores (QED and MCE-18). Protein structures were retrieved from the Protein Data Bank and preprocessed using AutoDockTools™, with docking simulations conducted via AutoDock Vina. The methodology incorporated a Lamarckian Genetic Algorithm combined with an exhaustive search strategy. In the course of the present study, 13 compounds were examined, and four of these were identified as leads: catechin, epicatechin, naringenin, and pinostrobin. This determination was made in accordance with the MPO criteria. These molecules exhibited both high permeability in Caco-2 and MDCK models and favorable hydration free energies (Δ*G*_hyd_ ≤ -5.0 kcal/mol). Futhermore, naringenin and pinostrobin were demonstrated to undergo metabolic transformation by CYP450 enzymes in hepatic microsomes, indicating limited metabolic stability. The docking results indicated strong binding affinities (*E*_A≤_ -6.0 kcal/mol) to CnFTase and AdSS, underscoring their potential as enzyme inhibitors through interactions within the active site, including residues associated with fluconazole binding site. Molecular dynamics simulations indicated a smaller conformational torsion of the Cα of the CnFTase and AdSS structures, suggesting that collective movements for both protein-ligand complexes are stable. The results suggest that these lead compounds are a starting point for new antifungal drugs inhibiting *C. neoformans*.

**Supplementary Information:**

The online version contains supplementary material available at 10.1007/s00203-026-04814-9.

## Introduction

There are more and more cases of infections caused by fungi, and they are becoming resistant to the medicines used to treat them. So, the World Health Organization (WHO) published the List of Priority Fungal Pathogens (LPPF) in October 2022. This is to encourage more research, surveillance, and policy interventions. The pathogens were grouped as critical, high, or medium priority. *Cryptococcus neoformans* was grouped as a “critical” fungal pathogen, where the classification criteria were based on factors such as resistance to antifungal drugs, mortality rates, how often it occurs each year, complications, long-term effects, and how it is spread around the world (Zhao et al. 2023).

*C. neoformans* is a fungus that has been responsible for numerous deaths, particularly among immunocompromised patients. It causes pulmonary fungal infections and cryptococcal meningitis, and it is the etiologic agent of cryptococcosis. This fungus can be found in bird droppings, decaying wood, and soil (Khan et al. 2024). *C. neoformans* is an opportunistic pathogen, and its transmission occurs through the air. Once the environment is contaminated, humans can inhale the dehydrated yeast spores and become infected, regardless of their immunity, posing a greater risk to people with compromised immune systems (Mackenzie and Klig 2008).

A recent study has identified a group of patients who are at an increased risk of complications from their underlying health condition. This group, which includes patients infected with HIV (human immunodeficiency virus), those undergoing cancer treatment (chemotherapy and immunotherapy), transplant patients, diabetics, individuals with liver or kidney disease, chronic lung disease, and viral infections of the respiratory tract, has been classified as a high-risk population. In the initial post-infection stage of the individual, colonization of the lungs is initiated, and if left untreated, can disseminate to the bloodstream, affecting other organs and the Central Nervous System (Tezcan et al. 2023). In addition to humans, *C. neoformans* has been documented to infect animals such as cats, horses, and cattle (Freitas et al. 2023).

The most common pharmaceutical agents employed in the treatment of cryptococcosis are fluconazole, amphotericin B, and echinocandins. The therapeutic approach entails the administration of a combination of these medications over an extended period, ranging from a few months to a year or more. However, with prolonged use, the drug can become toxic to the patient, and the fungus can develop resistance (Pereira de Sa et al. 2021). The elevated toxicity of pharmaceuticals has given rise to an increased demand for natural products. This phenomenon is further substantiated by studies that have demonstrated the presence of numerous bioactive molecules in plants, which exhibit a variety of biological activities, including antifungal properties (Thammasit et al. 2018).

From this perspective, *Anacardium occidentale L*., a member of the Anacardiaceae family, is commonly referred to as the cashew tree. This plant is native to Brazil and has been cultivated across the globe. A multitude of studies have demonstrated the presence of various secondary metabolites, including quercetin glycosides, condensed tannins, and phenolic acids, in diverse parts of the cashew tree (Konan and Bacchi 2007). The cashew tree has been shown to possess a wide range of biological activities, including bactericidal, fungicidal, vermicidal, amoebicidal, antioxidant, anti-inflammatory, and anti-ulcerogenic properties, as well as healing potential (Novaes and Novaes 2021).

Cashew nut kernels (CNA) are a foodstuff that is abundant in micronutrients and macronutrients, thus supplying the needs of the human body. In addition to this, cashew nut kernels contain phytochemical compounds and vitamins that have been shown to inhibit various diseases and strengthen the immune system. The compounds present in ACC have been identified as apigenin, catechin, epicatechin, galanin, kaempferol, naringenin, pinostrobin, and rutin (Woźniak et al. 2022). A variety of secondary metabolites have been identified in both raw and roasted ACC, including cardanol, cardol, and anacardic acids (monoene, diene, and triene), which have been documented in the extant literature to possess both antimicrobial and antioxidant properties (Trevisan et al. 2006).

In consideration of these findings, the objective of the present study was to conduct an in silico evaluation of the antifungal potential of the phytochemicals present in ACC against *C. neoformans*, employing a structure-based virtual screening approach.

## Materials and methods

### Multiparameter optimization

#### Structure complexity analysis

**Fig. 1:**
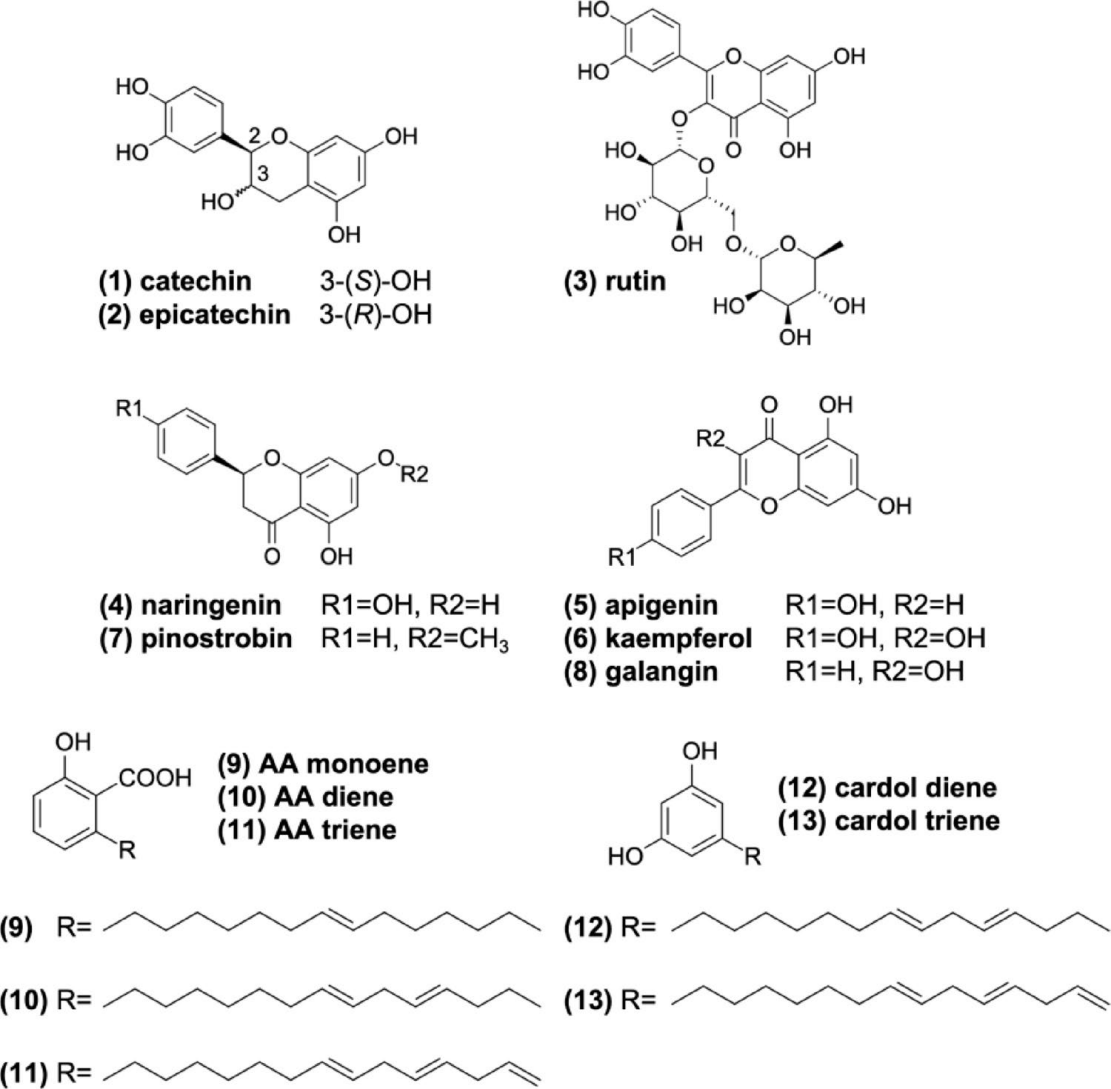
The two-dimensional representation of the structure of cashew nut (*Anacardium occidentale*) phytochemicals

The two-dimensional representation of the structure of cashew nut (*Anacardium occidentale*) phytochemicals (Fig. 1) was plotted and rendered using the academic license software MarvinSketch^®^ version 25.01.0, Chemaxon© (https://chemaxon.com/marvin), and converted into Simplified Molecular Line Entry System (SMILES) linear notation. This was then submitted to the ADMETlab 3.0 online server (https://admetlab3.scbdd.com/) for quantitative estimation of druglikeness (QED), as shown in Eq. 1:

1 $$\:QED=exp\left(\frac{{\sum\:}_{i=1}^{n}{w}_{i}ln{d}_{i}}{{\sum\:}_{i=1}^{n}{w}_{i}}\right)$$

where *w* is the weighting factor assigned to each physicochemical property *i*, ranging from 0.0 to 1.0 in relation to the desirability functions (*d*_i_), formed by the thresholds: intrinsic lipophilicity (logP) = 3, molecular weight (MW) = 360 g/mol, H-bond donors (HBD) = 1, H-bond acceptors (HBA) = 3, topological polar surface area (TPSA) = 50 Å², rotatable bonds (nRot) = 5, aromatic rings (AR) = 2, while the sum results in a QED score ranging from 0. 0 (poor similarity to the drug) to 1.0 (optimal similarity to the drug) (Bickerton et al. 2012). A quantitative estimation of structural complexity was then carried out using the Medicinal Chemistry Evolution 2018 (MCE-18) algorithm (Da Silva et al. 2023; Da Silva Lopes et al. 2024). The following formula was used to make the quantitative estimate of drug similarity:

2 $$ MCE18 = \left( {AR + NAR + Chiral + Spiro\frac{{Fsp3 + Cyc - Acyc}}{{1 + Fsp3}}} \right)Q $$

In this system of notation, “AR” denotes the number of aromatic rings, “NAR” denotes the number of non-aromatic or aliphatic rings, and “Fsp3” denotes the fraction of sp3 atoms distributed between the cyclic and acyclic portions. These parameters were evaluated based on current trends in medicinal chemistry, which emphasize the selection of larger, more polar compounds over those associated with intracellular toxicity (Ivanenkov et al. 2019). In order to establish a hierarchy of prioritized lead compounds, the resultant data were methodically categorized according to the following criteria:

- MCE-18 ≤ 45, signifying low three-dimensional complexity and traditional or widely recognized chemical structures.
- MCE-18 in the range of 45–63, denoting compounds exhibiting high similarity to molecules described in patents, thereby maintaining an optimal balance between structural complexity and synthetic feasibility.
- MCE-18 > 78, representing compounds with highly complex structures, yet with limited synthetic accessibility.

#### Molecular lipophilicity potential and hydration free energy

At this stage, the tool MarvinSketch^®^ version 25.01.0, Chemaxon© (https://chemaxon.com/marvin) was adjusted to apply a structural optimization using the Merck Molecular Force Field (MMFF94) method (Halgren 1996), ensuring the selection of the conformer with the lowest energy, for analysis of the molecular lipophilicity potential (MLP), as shown in the following formula:

3 $$\:MLP={\sum\:}_{i=1}^{N}{F}_{i}f\left({d}_{ik}\right)$$

In the context of molecular fragmentation, the total number of fragments is denoted by *N*, with *F*_i_ representing the lipophilic contribution of each fragment *i* of the molecule. *f*(*d*_i__k_), on the other hand, is a distance function dependent on the separation between fragment *i* and a point in *k*-space. Each point is conceptualized as a sphere, with the color denoting the specific region’s hydrophobicity. Specifically, blue signifies hydrophobic regions, while red indicates polar regions (Oberhauser et al. 2014). The outcomes were associated with the free energy of hydration (Δ*G*_hyd_), which is linked to lipophilicity in a relationship expressed by Eq. 4:

4 $$\Delta G_{{hyd}} {\text{ }} = {\text{ }}2.303RT \cdot \log P$$

where *R* is the ideal gas constant, *T* is the temperature, and 2.303 is the conversion factor for logP in natural logarithm (ln). Δ*G*_hyd_ values < −5.0 kcal/mol are associated with compounds with an ideal alignment between solubility and permeability (Zafar and Reynisson 2016).

In this study, specific cut-off values were applied to prioritize compounds with optimal drug-like characteristics based on established biopharmaceutical standards. Hydration free energy (ΔGhyd) values lower than − 5.0 kcal/mol were utilized as a key QSAR descriptor to identify compounds exhibiting an ideal alignment between aqueous solubility and membrane permeability, which is critical for ensuring efficient absorption and distribution (Zafar and Reynisson 2016; Mpiana et al. 2020).

### Pharmacokinetic prediction

The chemical structure of the ligands converted into SMILES inputs was subjected to a similarity test guided by artificial intelligence (AI). This similarity test was performed with compounds in the DrugBank^®^ database that show alignment between effective cell permeability (P_app, A→B_) and clearance in liver microsomes and in human and rat hepatocytes (Cl_int, u_). These compounds are related to in vivo pharmacokinetic properties such as human intestinal absorption (HIA) and oral bioavailability. The ADMET-AI predictor (https://admet.ai.greenstonebio.com/) was used to conduct this test (Da Rocha et al. 2022).

### Parallel artificial membrane permeability assay prediction

The AI-guided similarity test was adjusted to estimate the Parallel Artificial Membrane Permeability Assay (PAMPA) properties for P_app, A→B_ in cell lines of human colon epithelial cells (Caco-2) and Madin-Darby canine kidney cells (MDCK). A regression model was employed to utilize quantitative structure-property relationship (QSPR) data, thereby yielding logP_app, A→B_ values that were filtered by the biopharmaceutical classification system. Compounds exhibiting P_app, A→B_ values greater than 20 × 10^− 6^ cm/s were identified as those with high cell permeability and human intestinal absorption of approximately 96% (Wang et al. 2016).

### Metabolic stability estimation

The prediction of metabolic stability was carried out through a similarity test, based on linear regressions, with compounds present in the ChEMBL database (https://www.ebi.ac.uk/chembl/explore/document/CHEMBL3301361) with data from in vitro pharmacokinetic and metabolic stability tests (Hersey 2015), using the ADMET-AI predictor (https://admet.ai.greenstonebio.com/). The properties encompass lipophilicity at physiological pH (logD at pH 7.4), volume of distribution of steady state (V_dss_), and clearance descriptors in the liver microsome system (Cl_Micro_) and in hepatocytes (Cl_Hepa_) (human liver microsomes, human and rat hepatocytes). Compounds with Cl_Hepa_ < 100 µL/min/10^6^ cells (Cl_int, u_ < 100 mL/min/kg) are associated with metabolic stability values (Pettersson et al. 2016).

### Site of metabolism identification

The prediction of metabolic sites was conducted using the XenoSite tool (https://xenosite.org/), which generated a reactivity map that delineates the probability of compounds being substrates of xenobiotic metabolizing cytochrome P450 (CYP450) isoforms. This prediction was facilitated by the ProTox-II online server (https://tox.charite.de/protox3/). The tool’s functionality is enabled by a database that catalogs the sensitivity and structural specificity of known CYP450 substrates (Zheng et al. 2009). The results will be compared with the acute oral toxicity (LD_50_) predicted in rats using the Deep-PK tool (https://biosig.lab.uq.edu.au/deeppk/) (Lima et al. 2023).

### Ecotoxicity prediction

The compounds of interest, as represented by their SMILES structures, were subjected to a predictive ecotoxicity analysis using a systematic approach, employing the ADMETlab 3.0 platform (https://admetlab3.scbdd.com/). The evaluation encompassed parameters associated with organic toxicity and exposure toxicity. The following outcomes were subjected to analysis: The bioconcentration factor (BCF); the growth inhibition concentration (IGC_50_) in the aquatic protozoan *Tetrahymena pyriformis*; the lethal concentration (LC_50_) in *Pimephales promelas* (96-hour exposure) and *Daphnia magna* (48-hour exposure).

In addition to indicators of organic toxicity, such as cardiotoxicity, ototoxicity, dermal sensitization, carcinogenic potential, mutagenicity (Ames test), acute oral toxicity in rodents (ROA), and hepatotoxicity in humans (H-HT), were also considered. The investigation of exposure toxicity was conducted with parameters including eye corrosion (EC), eye irritation (EI), and neurotoxicity. The probability values obtained were then organized and visualized using heat maps, which were generated with the aid of the Morpheus statistical tool (https://software.broadinstitute.org/morpheus/) (Lopes et al. 2025).

### Molecular docking procedures

#### Enzyme preparation for docking studies

To study the mechanism of action of the phytochemicals present in cashew nut (*Anacardium occidentale*) against *C. neoformans*, molecular docking simulations were performed using the enzymes farnesyltransferase (CnFTase), beta-carbonic anhydrase (β-CA), and adenylosuccinate synthetase (AdSS) as targets. These enzymes were obtained from the Protein Data Bank (https://www.rcsb.org/) with PDB IDs: 7T08 (Wang et al. 2022), 2W3N (Schlicker et al. 2009), and 5I34 (Blundell et al. 2016) respectively. During the preparation of the targets, residues were removed, essential cofactors were maintained, and polar hydrogens and Kollman charges were added using the AutoDockTools™ software (https://autodocksuite.scripps.edu/adt/) (Yan et al. 2014).

#### Molecular docking simulation and data output

Molecular docking simulations were performed using the AutoDockVina software (Trott and Olson 2009), employing the Lamarckian Genetic Algorithm (LGA), with an exhaustiveness of 64 and a grid box covering the entire structure of the protein targets. The grid dimensions were as follows: for CnFTase, the axes 26.270 x, −32.730 y, −3.095 z and size 104 x, 121 y, 102 z; for β-CA, the axes 29.332 x, 16.424 y, 13.512 z and size 124 x, 82 y, 99 z; and for AdSS, the axes 21.975 x, 45.744 y, 27.115 z and size 85 x, 103 y, 114 z. For comparative data, simulations were also performed with Amphotericin B and Fluconazole. Fifty simulations were run, generating 20 poses per simulation. The best pose selection criterion was based on the statistical parameter Root Mean Square Deviation (RMSD) with values up to 2.0 Å and an affinity energy (*E*_A_) lower than − 6.0 kcal/mol (Yusuf et al. 2008; Shityakov and Förster 2014). The selection of molecular docking poses was based on an affinity energy (EA) threshold of ≤ −6.0 kcal/mol. This standard criterion was employed to ensure the selection of complexes with high binding stability and specificity, effectively distinguishing potent potential inhibitors from weak or non-specific binders (Yusuf et al. 2008; Shityakov and Förster 2014; Matondo et al. 2022).

### NMA-based MD simulation

Normal mode analysis (NMA) based on an elastic network model (ENM) was conducted to evaluate the conformational stability of the of the CnFTase receptor (PDB ID: 7T08) in complex with the lead compounds with best pharmacokinetic profile, and best molecular docking interaction, epicatechin, naringenin, and fluconazole (control); and for AdSS receptor (5I34) in complex with catechin and fluconazole (control) using the iMODS web server (https://imods.iqf.csic.es/), to assess the structural flexibility and stability of the receptor-ligand complex obtained in the molecular docking simulations. The PDB complex was loaded into iMODS, where NMA was performed to evaluate the conformational MD of the complex. The stability of the complex is indicated by the relationships between RMSD Cα and iteration index (Gaillard et al. 1994; López-Blanco et al. 2014; Gonçalves et al. 2026).

## Results and discussion

### Multiparameter optimization

#### Structure complexity analysis

**Fig. 2:**
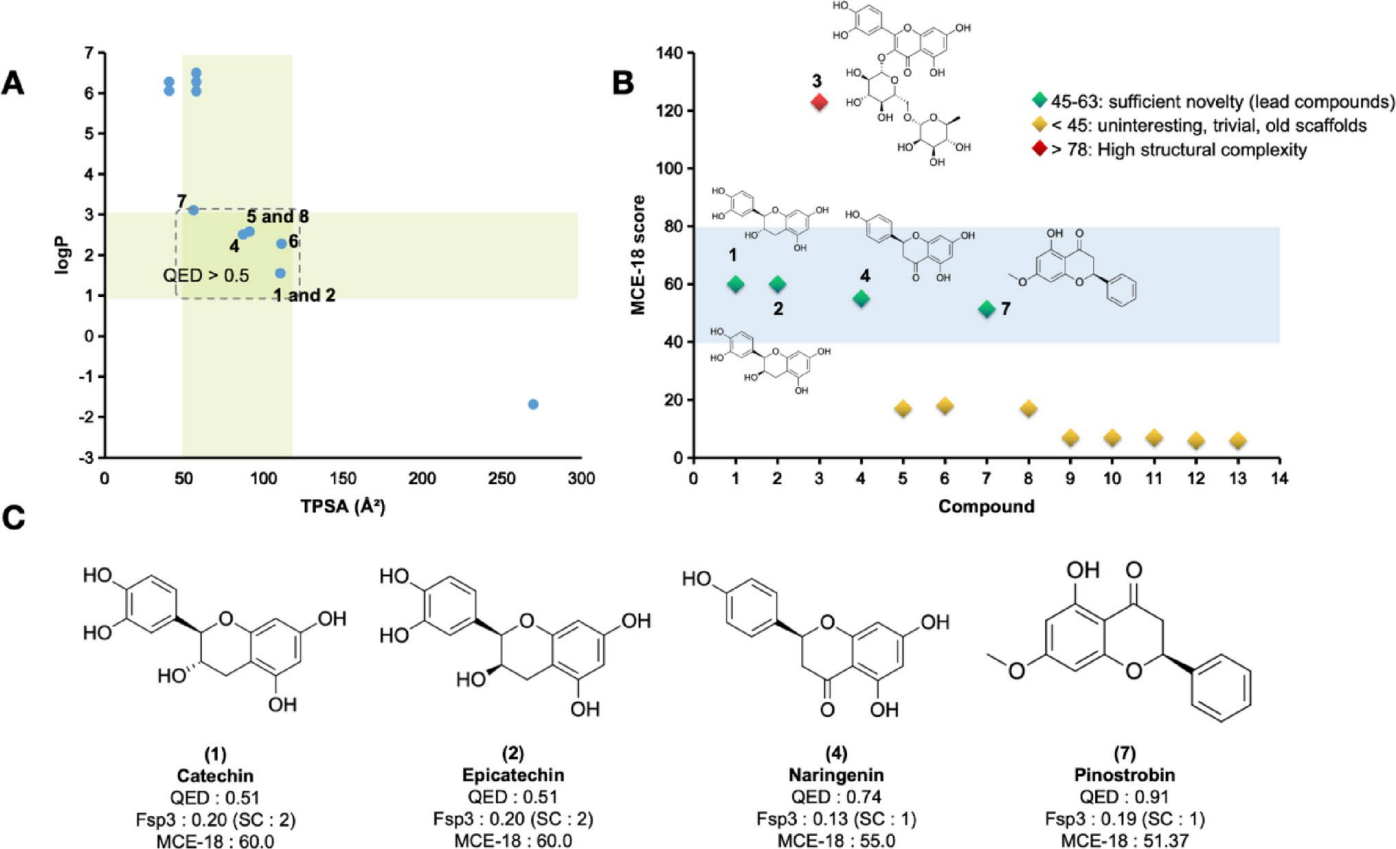
**A** Estimation of QED score in relation to the alignment between lipophilicity (logP) and polarity (TPSA) of cashew nut phytochemicals, **B** prediction of structural complexity dependent on MCE-18 score, and **C** relationship between QED and MCE-18 scores calculated in relation to Fsp3

Using a multiparametric optimization (MPO) analysis, it was possible to make a quantitative estimation of druglikeness (QED) of the phytochemicals present in the almond of *A. occidentale*. Initially, 7 compounds with QED scores ≥ 0.5 were filtered out, on a scale ranging from 0.0 to 1.0, which include (1) Catechin, (2) Epicatechin, (4) Naringenin, (5) Apigenin, (6) Kaempferol, (7) Pinostrobin, and (8) Galangin (Table 1). Interestingly, a structural trend is observed that focuses on the selection of more lipophilic compounds (logP 1–3) that are more polar, due to the presence of H-bond donor (HBD) groups, i.e. whose calculated TPSA values are between 50 and 120 Å², a chemical singularity related to compounds with therapeutic action that have been registered in patents in recent years (Fig. 2a) (Ivanenkov et al. 2019). Compounds within this physicochemical threshold exhibit a superior alignment between elevated cell permeability (P_app, A→B_ > 10 × 10^− 6^ cm/s) and diminished intrinsic hepatic clearance (Cl_int, u_ < 8.0 mL/min/kg), as defined by the Pfizer, Inc. biopharmaceutical classification system (Pettersson et al. 2016).

**Table 1:** Physicochemical properties calculated and applied to the quantitative druglikeness estimation (QED) criterion

Compound		logP	MW (g/mol)	HBA	HBD	TPSA (Å²)	nRot	nRing	QED
1	Catechin	1.55	290.08	6	5	110.38	1	3	0.510
2	Epicatechin	1.55	290.08	6	5	110.38	1	3	0.510
3	Rutin	–1.69	610.15	16	10	269.43	6	5	0.140
4	Naringenin	2.51	272.07	5	3	86.99	1	3	0.742
5	Apigenin	2.58	270.05	5	3	90.9	1	3	0.632
6	Kaempferol	2.28	286.05	6	4	111.13	1	3	0.546
7	Pinostrobin	3.11	270.09	4	1	55.76	2	3	0.911
8	Galangin	2.58	270.05	5	3	90.9	1	3	0.632
9	AA monoene	6.5	346.25	3	2	57.53	14	1	0.298
10	AA diene	6.28	344.24	3	2	57.53	13	1	0.327
11	AA triene	6.05	342.22	3	2	57.53	13	1	0.339
12	cardol diene	6.28	316.24	2	2	40.46	12	1	0.352
13	cardol triene	6.06	314.22	2	2	40.46	12	1	0.363

*MW* Molecular weight, *HBA* H-bond acceptors, *HBD* H-bond donors, *TPSA* Topological polar surface area, *QED* Quantitative estimation of druglikeness

Conversely, when assessing the degree of structural complexity, it was observed that (3) Rutin may exhibit a limited pharmacokinetic profile, particularly due to its high polarity (TPSA > 200.00 Å²) (Table 1), in addition to possessing two highly torsionable glycoside fragments (nRot = 6) with at least 10 steric centers (Table 2), resulting in an MCE-18 score > 78.0, indicating a high structural complexity (Fig. 2b). Conversely, the ligands (1) catechin, (2) epicatechin, (4) naringenin, and (7) pinostrobin demonstrate an alignment between Fsp3 (> 0.10) and stereo centers (SC ≤ 2). This is evidenced by MCE-18 scores ranging from 50.0 to 60.0, indicative of a substantial degree of structural novelty. This novelty enables optimization of pharmacokinetic/pharmacodynamic properties and synthetic accessibility (Fig. 2c). As a result, these compounds are classified as leading compounds.

Furthermore, compounds classified as MCE-18 ≥ 50.0 are considered promising lead compounds for membrane receptor modulators, protease inhibitors, and ion channel ligands. These compounds exhibit low affinity for kinases (Ivanenkov et al. 2019).

**Table 2:** Structural complexity properties, expressed in Fsp3 and stereo centers (SC), and lipophilicity descriptors expressed in logD and free energy of hydration (Δ*G*_hyd_)

Compound	ΔG_hyd_ (kcal/mol)	Fsp3	SC	MCE-18	logD
Catechin	**−13.11**	**0.20**	**2**	**60.00**	**0.95**
Epicatechin	**−13.22**	**0.20**	**2**	**60.00**	**0.95**
Rutin	−15.67	0.44	10	122.95	0.77
Naringenin	**−10.59**	**0.13**	**1**	**55.00**	**2.35**
Apigenin	−12.31	0.00	0	17.00	2.67
Kaempferol	−12.45	0.00	0	18.00	2.29
Pinostrobin	**−8.80**	**0.19**	**1**	**51.37**	**3.3**
Galangin	−11.92	0.00	0	17.00	2.71
AA monoene	−4.86	0.59	0	7.00	3.08
AA diene	−5.39	0.50	0	7.00	2.97
AA triene	−5.58	0.41	0	7.00	3.01
cardol diene	−3.86	0.52	0	6.00	4.84
cardol triene	−4.10	0.43	0	6.00	4.77

Highlighted in bold the lead compounds filtered by multiparametric optimization

#### Molecular lipophilicity potential and hydration free energy

Molecular lipophilicity potential (MLP) analysis can provide a topological view of the alignment between lipophilicity and polarity, attributes often associated with the pharmacokinetic potential in the lead compounds, such as the ability to permeate cellular lipid bilayers and metabolic stability (Gaillard et al. 1994; Pettersson et al. 2016). Consequently, the free energy of hydration (Δ*G*_hyd_) emerges as a QSAR descriptor, correlating the lipophilicity and polarity of a drug with its degree of aqueous solubility. This relationship is pivotal for understanding the absorption, permeability, and distribution of these compounds (Zafar and Reynisson 2016). Consequently, a Δ*G*_hyd_ of less than **−** 5.0 kcal/mol is indicative of compounds exhibiting enhanced lipophilicity-solubility efficiency.

**Fig. 3:**
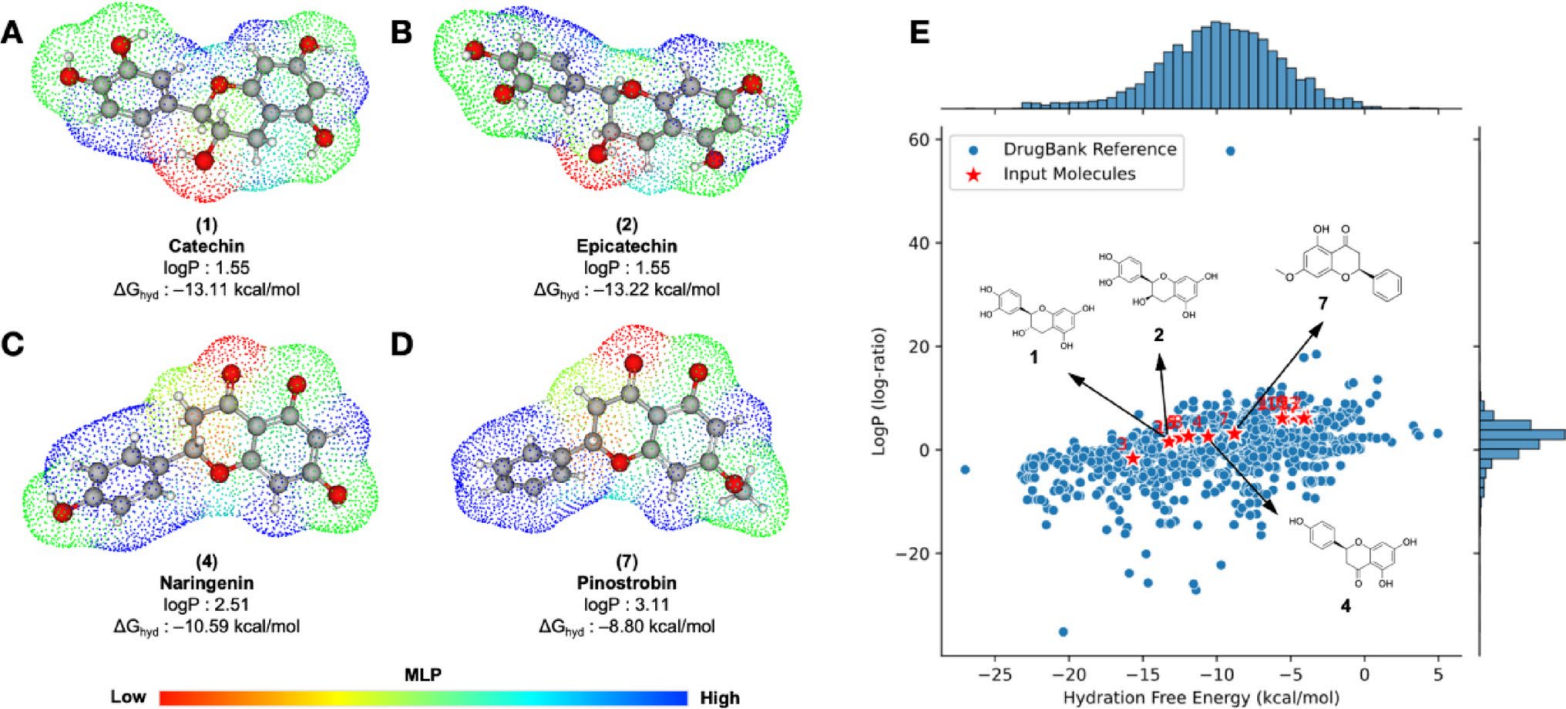
Surface analysis of the molecular lipophilicity potential (MLP) of lead compounds **A** catechin, **B **epicatechin, **C** naringenin and **D** pinostrobin, and **E** AI-guided similarity test for estimation of Δ*G*_hyd_

In the course of analyzing the molecular-level properties (MLP) of the lead compounds, it was observed that the catechol ring present in the compounds (1) catechin (Fig. 3a) and (2) epicatechin (Fig. 3b) strongly contributes to the formation of more water-soluble analogues (blue to green spectra). This is due to the greater predominance of –OH groups as HBD, which results in the formation of more polar (TPSA_R–OH_ = 20.23 Å²) and less fat-soluble compounds (logP = 1.55) (Ertl 2007). The decrease in these HBD groups is accompanied by the emergence of a more hydrophobic region in the B ring of the analogues (4) Naringenin and (7) Pinostrobin (blue color spectra), while the hydrophilic surfaces are concentrated in the heterocyclic ring (yellow to red spectra). This results in logP in the order of 2.51 for Naringenin (Fig. 3c) and in the order of 3.11 for Pinostrobin (Fig. 3d).

In an AI-guided similarity test, it is possible to observe a physicochemical trend where the change in Gibbs free energy (Δ*G*_hyd_) increases in an order directly proportional to the intrinsic lipophilicity (logP). In this test, it was observed that (7) Pinostrobin presented a Δ*G*_hyd_ value of **-**8.80 kcal/mol, within a percentile threshold of 58.51%, which indicates the degree of data similarity in relation to the compounds deposited in DrugBank^®^ (Fig. 3e) (Swanson et al. 2024). Compounds within this Δ*G*_hyd_ range (between **−** 10.0 and **−** 5.0 kcal/mol) exhibit an alignment between solubility and permeability.

### Parallel artificial membrane permeability assay prediction

**Fig. 4:**
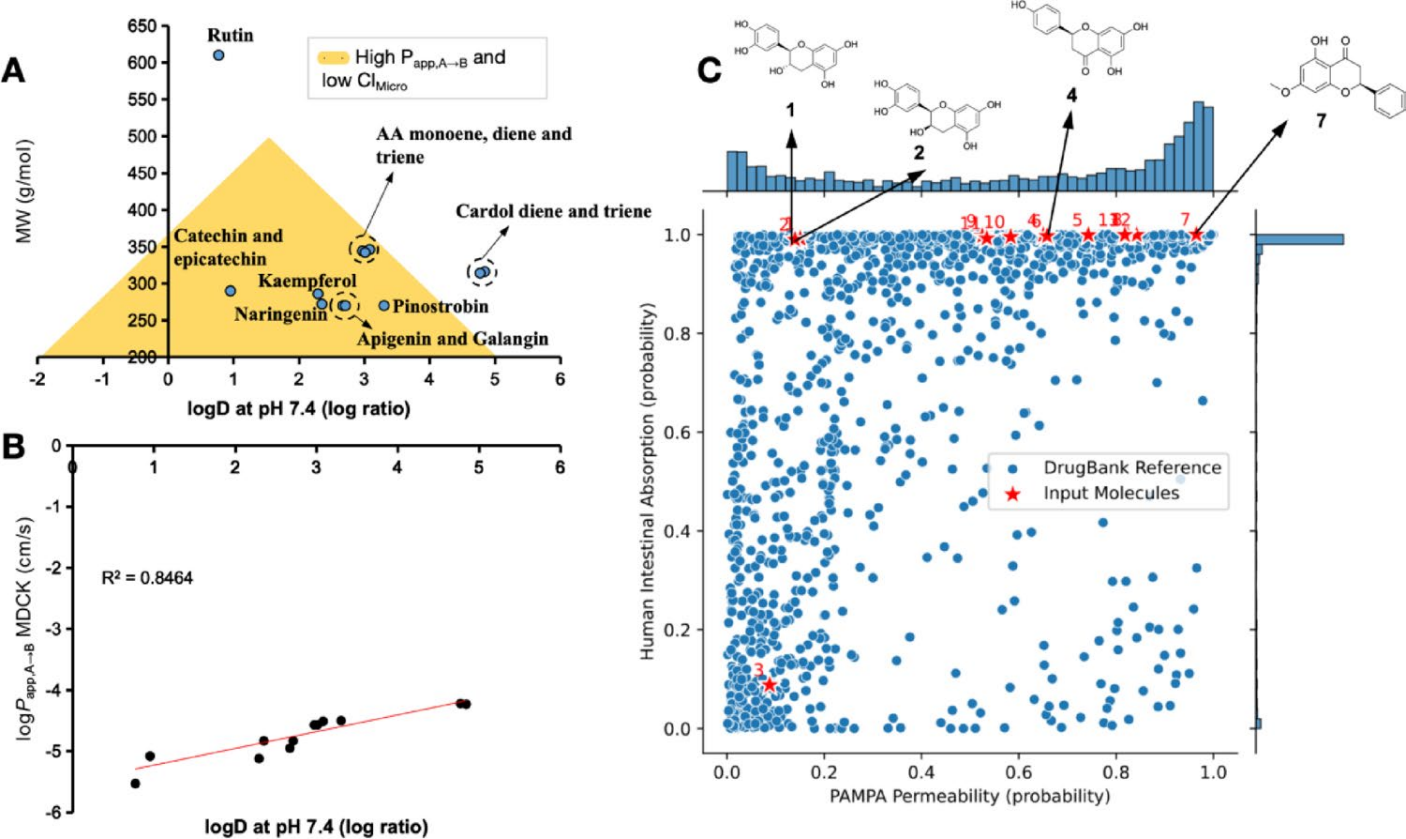
**A** Alignment between pH-dependent lipophilicity (logD at pH 7.4) and molecular weight (MW) for estimation of PAMPA profile for passive cell permeability (P_app, A→B_) and clearance in liver microsomes (Cl_Micro_), **B** correlation between log P_app, A→B_ MDCK and logD at pH 7.4 and **C** AI-guided similarity test for estimation of PAMPA profile in relation to human intestinal absorption

The PAMPA-based ADMET prediction model was employed to estimate the in vitro pharmacokinetic profile of phytochemicals (Sun et al. 2017; Rácz et al. 2023). It was observed that the ligands cardol diene and cardol triene exhibited logD values greater than 4.0 (log-ratio), a consequence of their extended alkyl moiety and diminished polarity (TPSA = 40.46 Å²). This property may, in turn, result in a reduction of their metabolic stability (Fig. 4a) (López-Blanco et al. 2014). Conversely, rutin, a phytochemical characterized by its high molecular weight (MW) of greater than 600 g/mol, as depicted in Fig. 4a, may potentially attenuate its PAMPA profile. The golden region of the triangle indicates the compounds with alignment between low hepatic clearance and favorable cellular permeability in relation to the Pfizer, Inc. database (Johnson et al. 2009).

It is noteworthy that MDCK permeability indices (in log P_app, A→B_ MDCK) exhibit a direct proportional increase in conjunction with the rise in clogD, exhibiting a correlation coefficient of R² = 0.8464. The log P_app, A→B_ MDCK values are predominantly concentrated within the range of − 5.0 to − 4.2 cm/s, as depicted in Fig. 4b (Leung et al. 2016). Furthermore, the AI-guided similarity test demonstrated that, with the exception of compound (3) rutin, the phytochemicals exhibited optimal human intestinal absorption (HIA), attributable to the PAMPA profile based on elevated cellular permeability, with P_app, A→B_ Caco-2 values greater than 2.0 × 10^–6^ cm/s (Fig. 4c).

In this study, compound (7) Pinostrobin demonstrated a notable profile, exhibiting an estimated P_app, A→B_ Caco-2 value of 2.29 × 10^–5^ cm/s and a P_app, A→B_ MDCK of 3.16 × 10^–5^ cm/s. These findings suggest that the compound possesses the capacity to permeate even the most selective biological membranes (Ma et al. 2005; Wager et al. 2016). Among the other leading compounds, it is evident that compounds (1) catechin, (2) epicatechin, and (4) naringenin exhibit lower P_app, A→B_ Caco-2 and MDCK, yet remain optimized for these cell lines, suggesting a pharmacokinetic profile characterized by high HIA and adequate oral bioavailability (see Table 3) (Pires et al. 2018).

**Table 3:** Prediction of PAMPA descriptors expressed in cell permeability (P_app, A→B_) in Caco-2 and MDCK cell lines, and metabolic stability descriptors expressed in intrinsic hepatic clearance (Cl_int, u_), clearance in hepatic microsomes (Cl_Micro_), clearance in cellular hepatocytes (Cl_Hepa_), and volume of distribution of sediment (V_dss_)

Compound		*P*_app, A→B_ Caco-2 (cm/s)	*P*_app, A→B_ MDCK (cm/s)	Cl_int, u_ (mL/min/kg)	Cl_Micro_ (µL/min/mg)	Cl_Hepa_ (µL/min/10^6^ cells)	Vdss (L/kg)
1	Catechin	**1.91 × 10** ^**−6**^	**8.32 × 10** ^**−6**^	**13.6**	**−17.04**	**53.07**	**1.17**
2	Epicatechin	**1.91 × 10** ^**−6**^	**8.32 × 10** ^**−6**^	**13.59**	**−16.11**	**53.80**	**1.12**
3	Rutin	2.51 × 10^− 7^	2.95 × 10^− 6^	13.3	40.10	25.57	0.87
4	Naringenin	**2.00 × 10** ^**−5**^	**1.48 × 10** ^**−5**^	**8.89**	**13.94**	**105.57**	**0.98**
5	Apigenin	1.12 × 10^− 5^	1.12 × 10^− 5^	4.07	7.99	92.70	0.35
6	Kaempferol	5.50 × 10^− 6^	7.59 × 10^− 6^	5.81	6.22	86.24	0.15
7	Pinostrobin	**2.29 × 10** ^**−5**^	**3.16 × 10** ^**−5**^	**7.91**	**47.28**	**127.09**	**1.48**
8	Galangin	1.10 × 10^− 5^	1.48 × 10^− 5^	2.5	23.75	101.14	0.30
9	AA monoene	9.12 × 10^− 6^	3.09 × 10^− 5^	3.58	59.59	97.06	0.25
10	AA diene	9.12 × 10^− 6^	2.69 × 10^− 5^	3.38	59.97	98.98	0.32
11	AA triene	1.00 × 10^− 5^	2.69 × 10^− 5^	2.95	51.87	88.19	0.54
12	cardol diene	1.07 × 10^− 5^	5.89 × 10^− 5^	−0.18	91.30	128.93	3.63
13	cardol triene	1.23 × 10^− 5^	6.03 × 10^− 5^	−0.29	83.01	118.14	5.37

Highlighted in bold the lead compounds filtered by multiparametric optimization

### Metabolic stability estimation

The present study examined the metabolic stability of a series of 13 cashew nut phytochemicals by predicting microsomal hepatic clearance (Cl_Micro_) and hepatocyte clearance (Cl_Hepa_). As illustrated in Table 3, a comparison of the estimated values reveals that Cl_int, u_ rates lower than 8.0 mL/min/kg are indicative of phase I of hepatic metabolism having minimal effect on the oral bioavailability of the compounds (5) Apigenin, (6) Kaempferol, (7) Pinostrobin, (8) Galangin, (9) AA monoene, (10) AA diene, and (11) AA triene (Zamek-Gliszczynski et al. 2011).

Negative prediction values refer to outliers resulting from low data similarity with compounds present in the DrugBank^®^ database, adjusted from AI-guided prediction (Table 3) (Swanson et al. 2024). In accordance with the prevailing consensus, Cl_Micro_ values for compounds (4) naringenin, (5) apigenin, (6) kaempferol, (7) pinostrobin, and (8) galangin were estimated to be lower than 40 µL/min/mg. This finding suggests that these compounds exhibit greater stability in liver microsomes than in hepatocytes. The calculated Cl_Hepa_ values for hepatocytes were found to be greater than 80 µL/min/10^6^ cells, indicating a higher level of stability in hepatocytes.

Among the compounds under consideration, a V_dss_ of approximately 1.48 L/kg is estimated for pinostrobin, particularly due to its moderately high lipophilicity (clogP > 3.0). This lipophilicity may affect the metabolic stability of the compound and increase its distribution in biological tissues (Table 3) (Pires et al. 2018). However, the compounds cardol diene and cardol triene were identified as the most lipophilic compounds in this study. Nevertheless, they are outside the threshold of the leading compounds filtered by MPO analysis. In conjunction with the leading compounds, the compound exhibits a PAMPA range that exceeds 2.0 × 10^− 5^ cm/s, indicative of a moderate to high permeability range (see Table 3).

### Site of metabolism identification

A substantial body of research has demonstrated that a considerable number of adverse drug reactions (ADRs) subsequent to phase I metabolism are attributable to electrophilically reactive metabolites that conjugate to nucleophilic sites in deoxyribonucleic acid (DNA) and proteins. These reactions have been shown to elicit toxic immune responses, drug-induced liver injury (DILI), and mutagenicity, in addition to affecting oral bioavailability and daily oral dose (Yu et al. 2014; Hughes et al. 2015; Hughes and Swamidass 2017). In this instance, a prediction model utilizes structure-property relationship (SPR) descriptors derived from an alignment between structural specificity and sensitivity to first-pass, CYP450-dependent, and second-pass, conjugation-dependent biotransformation (Zheng et al. 2009).

**Fig. 5:**
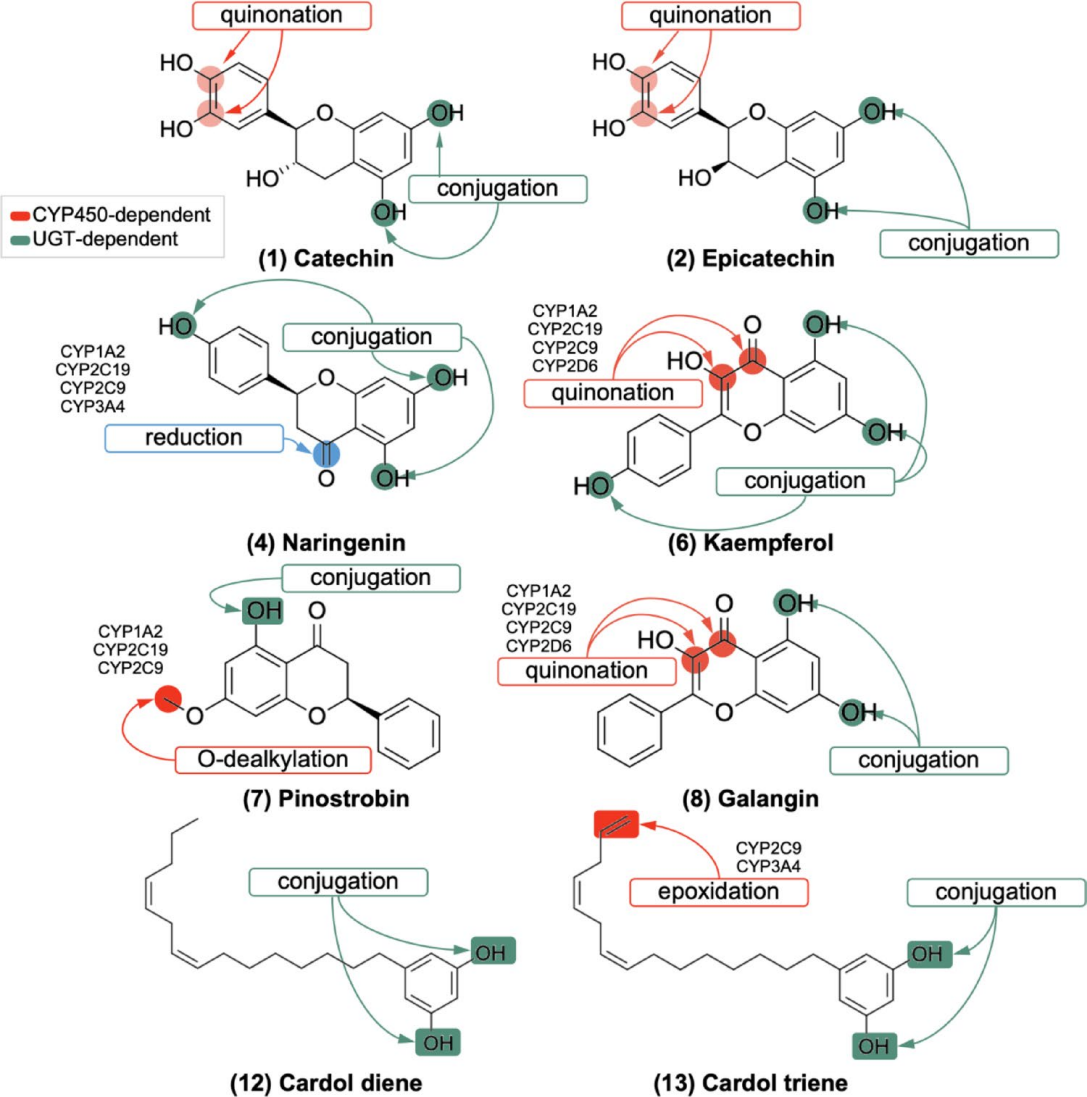
Metabolism site prediction for compounds likely to be substrates for CYP450 isoforms: (1) catechin, (2) epicatechin, (4) naringenin, (6) kaempferol, (7) pinostrobin, (8) galangin, (12) cardol diene and (13) cardol triene

The results indicated the presence of quinonation sites within the catechol ring of compounds (1) catechin and (2) epicatechin, with a probability lower than 0.7, yet higher than 0.7 in the conjugated system of the dihydropyran heterocyclic ring in compounds (6) kaempferol and (8) galangin, primarily attributable to the isoforms of CYP1A2, CYP2C19, CYP2C9, and CYP2D6 (Fig. 5). This specific site has been shown to induce a toxic response by metabolic activation, as it results in the formation of electrophilic products that have the capacity to covalently interact with DNA nucleotides (Hughes and Swamidass 2017).

The structural specificity of compounds (4) naringenin and (7) pinostrobin reveals their susceptibility to being metabolized by reduction and O-dealkylation, respectively, depending on isoforms such as CYP1A2, CYP2C19, and CYP2C9 (Fig. 5). Conversely, (13) Cardol triene exhibits a terminal alkene that functions as a substrate for CYP3A4. This reacts with the formation of an epoxide-based metabolite, which subsequently serves as an intermediate in an aliphatic hydroxylation process. This hydroxylation process has the capacity to be reactive against proteins and DNA (Fig. 5).

In summary, the LD_50_ values for compounds with a lower propensity to generate reactive metabolites in the liver microsome system were predicted to exceed 2.0 mmol/kg. Conversely, the derivatives naringenin, pinostrobin, cardol diene, and cardol triene exhibited estimated acute toxicity values of 1.99 (Table 4). The LD_50_ of naringenin is 1.99 mmol/kg, and it is listed among the leading compounds (Table 4). UDP-glucuronosyltransferase (UGT)-dependent O-conjugation is the predominant metabolic process for naringenin. The range of positive LD_50_ values expressed in log_10_(mmol/kg) indicates that the compounds are low in susceptibility to result in acute oral toxicity by metabolic activation (Lagunin et al. 2011).

**Table 4:** Estimated descriptors of acute toxicity in rats (LD_50_), bioconcentration factor, chronic toxicity in Fathead Minnow (96 h), acute toxicity in *Daphnia magna* (48 h), and growth inhibition of *Tetrahymena pyriformis*

Compounds	LD50 (log_10_ [mmol/kg])	BCF (log_10_[L/kg])	LC_50_ FM −log_10_[(mg/L)]	LC_50_ DM −log_10_[(mg/L)]	IGC_50_ TP −log_10_[(mg/L)]
Catechin	2.01	0.75	2.90	4.13	3.77
Epicatechin	2.01	1.01	3.13	4.26	3.91
Rutin	2.21	0.53	3.18	4.68	3.93
Naringenin	1.99	1.25	3.71	4.45	4.02
Apigenin	2.14	1.31	3.69	4.35	3.99
Kaempferol	2.46	1.24	3.65	4.38	4.01
Pinostrobin	1.84	1.62	4.05	5.51	4.75
Galangin	2.47	1.15	4.10	4.84	4.53
AA monoene	2.67	1.03	4.49	5.91	3.51
AA diene	2.63	1.25	4.54	5.90	4.01
AA triene	2.64	1.41	4.55	5.93	4.29
cardol diene	1.75	1.66	4.92	6.01	5.60
cardol triene	1.93	1.86	4.83	5.99	5.57

### Ecotoxicity prediction

The ecotoxicity of chemicals dispersed in the air and water can be directly related to the mortality of animal species in the impacted ecosystem. Determining the concentration capable of inducing lethality in a fish population within a specified period of 96 h can provide relevant data on the bioconcentration factor of low molecular weight organic compounds. This factor is a critical parameter for evaluating the distribution of these chemicals between the atmosphere and the aquatic environment (Hayyan et al. 2024). An analysis of the environmental toxicity spectrum revealed that the compounds exhibited bioconcentration factor (BCF) indices below 2.0 L/kg, suggesting minimal propensity for accumulation within cells or cell species (see Table 4).

**Fig. 6:**
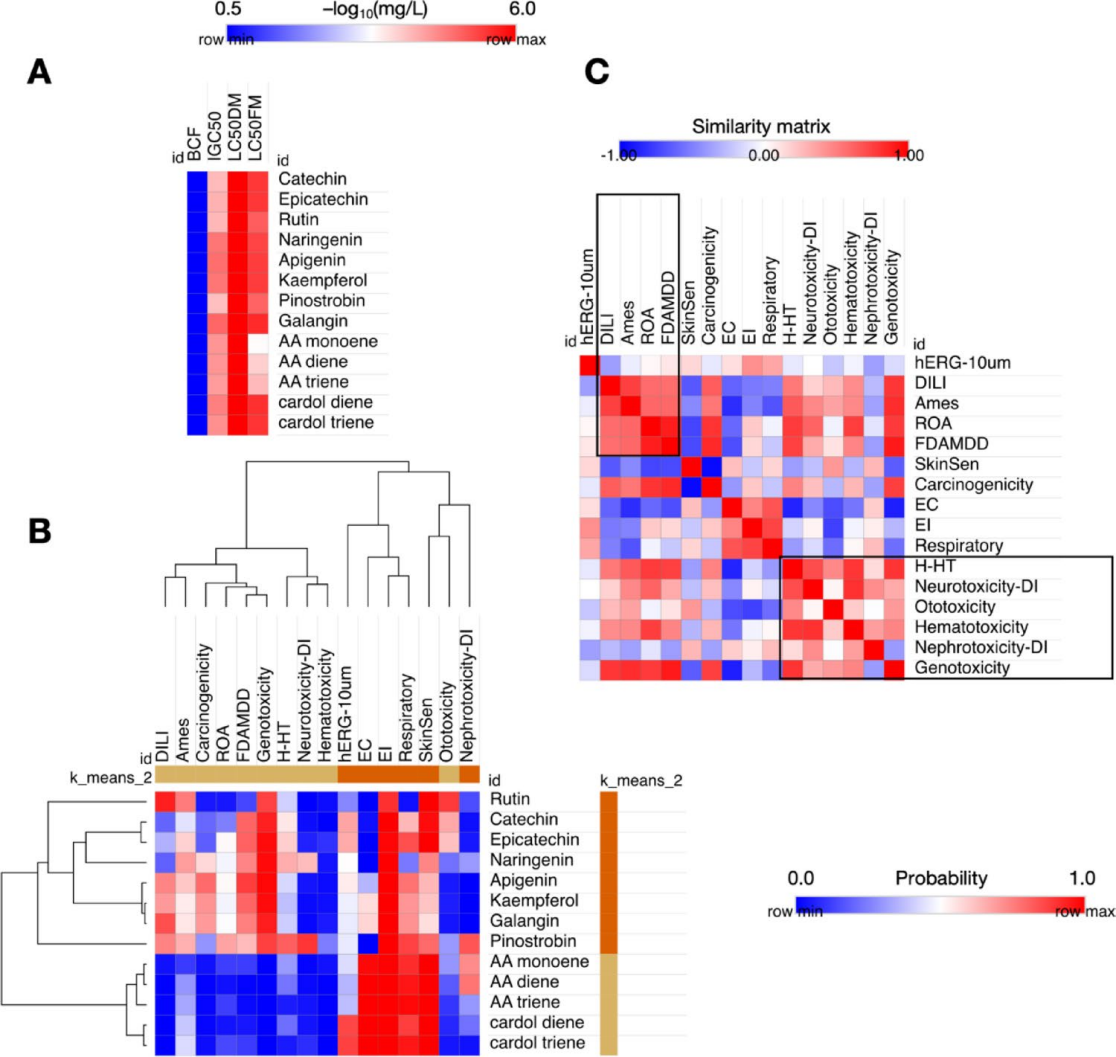
**A** Prediction of bioconcentration factor (BCF), chronic toxicity spectrum in Fathead Minnow (96 h), acute toxicity in *Daphnia magna* (48 h), and growth inhibition of *Tetrahymena pyriformis*, **B** Prediction of probability of organic toxic response and ecotoxicity, and **C** similarity matrix between toxicity descriptors

Conversely, the LC_50_ heatmap exhibited a pronounced propensity for the compounds to induce lethality in 50% of the aquatic species, specifically the Fathead Minnow (96 h) and the *Daphnia magna* (48 h). The values are expressed in an order greater than 2.00 in -log_10_(mg/L). As illustrated in Fig. 6a, the data indicate that a concentration greater than 3.0 in -log_10_(mg/L) corresponds to an inhibitory concentration value (IGC_50_) for the species *Tetrahymena pyriformis*. This finding suggests that environmental species may exhibit an acute or chronic toxic response to exposure (Hayyan et al. 2024).

When analyzing the probability of phytochemicals inducing organic toxicity and toxicity by exposure, it is possible to observe a hierarchical order between the ligands and the toxicity descriptors (Fig. 6b). In this predictive test, it is possible to observe that the most lipophilic analogues, that is, the derivatives AA monoene, AA diene, AA triene, cardol diene and cardol triene, have a high probability of causing ocular toxicity (EC - eye corrosion and EI - eye irritation), respiratory toxicity and skin sensitization (probability greater than 9.0), while in the compounds of less structural complexity, that is, catechin, epicatechin, naringenin, apigenin, kaempferol, galangin and pinostrobin, indicators of organic toxicity predominate (Fig. 6b).

In this study, pinostrobin demonstrated a probability greater than 0.6 of inducing liver damage (DILI and H-HT descriptors), mutagenic response (Ames descriptor), and genotoxicity. Conversely, naringenin was shown to be less toxic to the human liver when these compounds were compared (Fig. 6b). A comparison of the toxicity descriptors in the similarity matrix reveals a higher prevalence of organic toxicity models among the ligands, particularly those associated with DILI, mutagenicity, neurotoxicity, and acute toxicity, including rat oral acute (ROA) toxicity and FDA Maximum Daily Dose (FDAMDD). This observation suggests the potential for restricting the daily oral dose of compounds to ≤ 0.011 mmol/kg-bw/day (Fig. 6c).

### Molecular docking of the cashew nut phytochemicals

We conducted a series of computational simulations using the molecular docking technique to investigate the mechanism of action of phytochemicals present in cashew nut kernel (ACC) against farnesyltransferase (CnFTase), β-carbonic anhydrase (β-CA), and adenylosuccinate synthetase (AdSS). The molecular docking study demonstrated that the compounds exhibited RMSD values within the range of 2.0 Å, as reported in the extant literature (Yusuf et al. 2008) (see supplementary tables S1, S2, and S3). This finding substantiates the feasibility of receptor-ligand complex formation. The data also demonstrate a favorable affinity energy for the complexes formed.

**Fig. 7:**
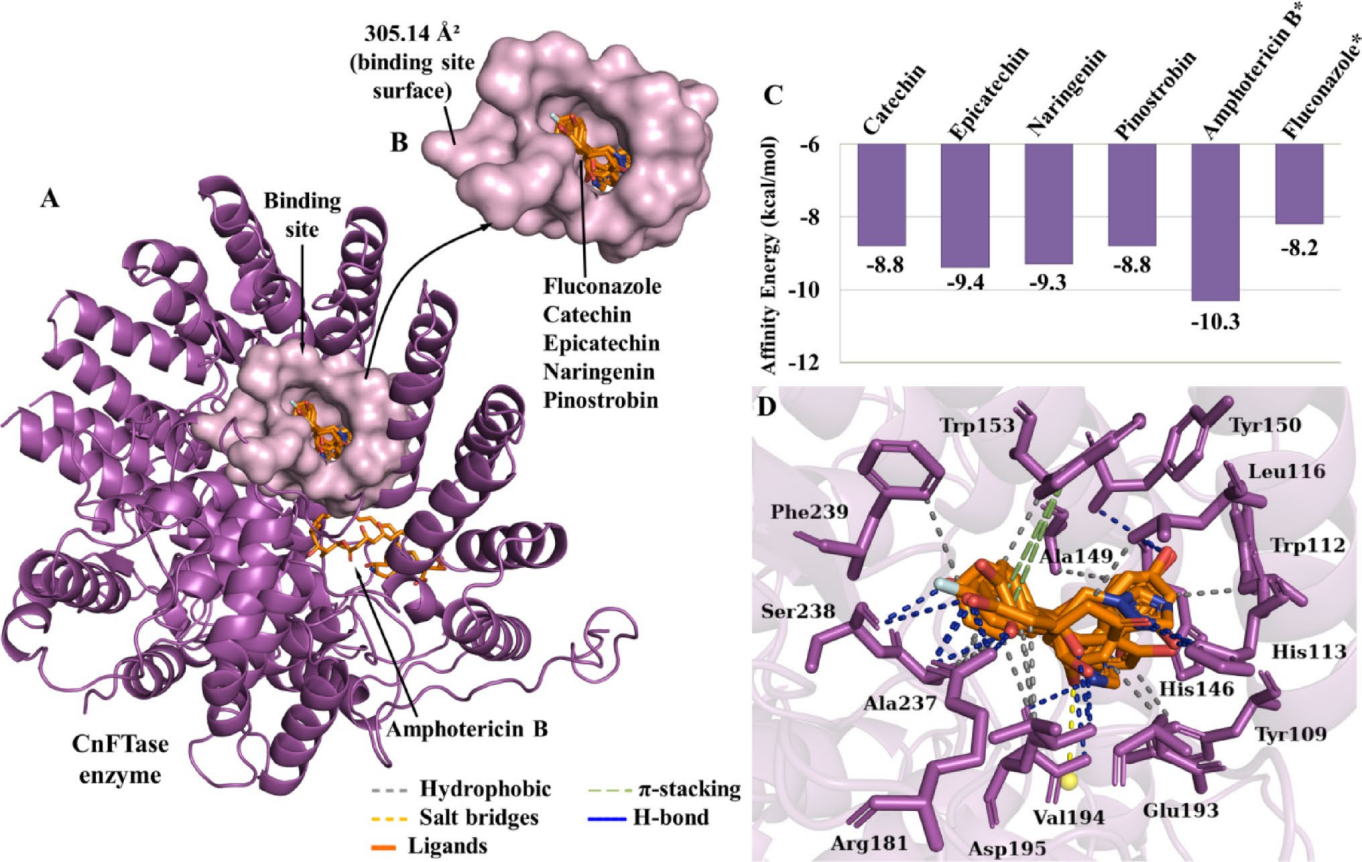
**A** Three-dimensional image of the CnFTase protein, **B** the binding cavity, **C** affinity energy (kcal/mol) of the leads compounds and controls*, **D** binding site of fluconazole and lead compounds

In the context of CnFTase (Fig. 7a), the four compounds that demonstrated high pharmacokinetic viability (lead compounds) were identified, namely catechin, epicatechin, naringenin and pinostrobin. The controls amphotericin B, and fluconazole presented *E*_A_ equal to −10.3 kcal/mol and − 8.1 kcal/mol, respectively (Fig. 7c), which are within the expected affinity threshold (Shityakov and Förster 2014). The molecular docking results demonstrated that the lead compounds formed complexes in the same binding site as the fluconazole control (Fig. 7d). The predicted protein cavity has a molecular surface area of ​​305.14 Å^2^ (Fig. 7b) resulting from the side chain of amino acid residues, which include Tyr109A, Trp112A, His113A, Leu116A, Tyr145A, His146A, Ala149A, Tyr150A, Trp153A, Arg181B, Glu193B, Val194B, Asp195B, Phe223B, Cys236B, Ala237B, Ser238B, Phe239B, Pro240B, Met262B and Tyr269B, where the predominance of apolar and aromatic side chain residues constitute a hydrophobic cavity.

The analysis of ligand-CnFTase interactions (Supplementary Table S1) suggests that the phytochemicals showed a series of interactions similar to the fluconazole control, especially with residues Trp112A, His113A, Ala149A, Trp153A, Arg181B, Glu193B, Asp195B, Ala237B, (catechin); His146A, Tyr150A, Arg181B, Val194B, Ala237B, Ser238B (epicatechin); His146A, Trp153A, Arg181B, Val194B, Asp195B, Ala237B, Ser238B (naringenin); Tyr109A, His113A, Leu116A, Val194B, Ala237B, Phe239B (pinostrobin), indicating similar action to the drug used (Fig. 7D), these being the lead compounds with the greatest specificity for CnFTase.

**Fig. 8:**
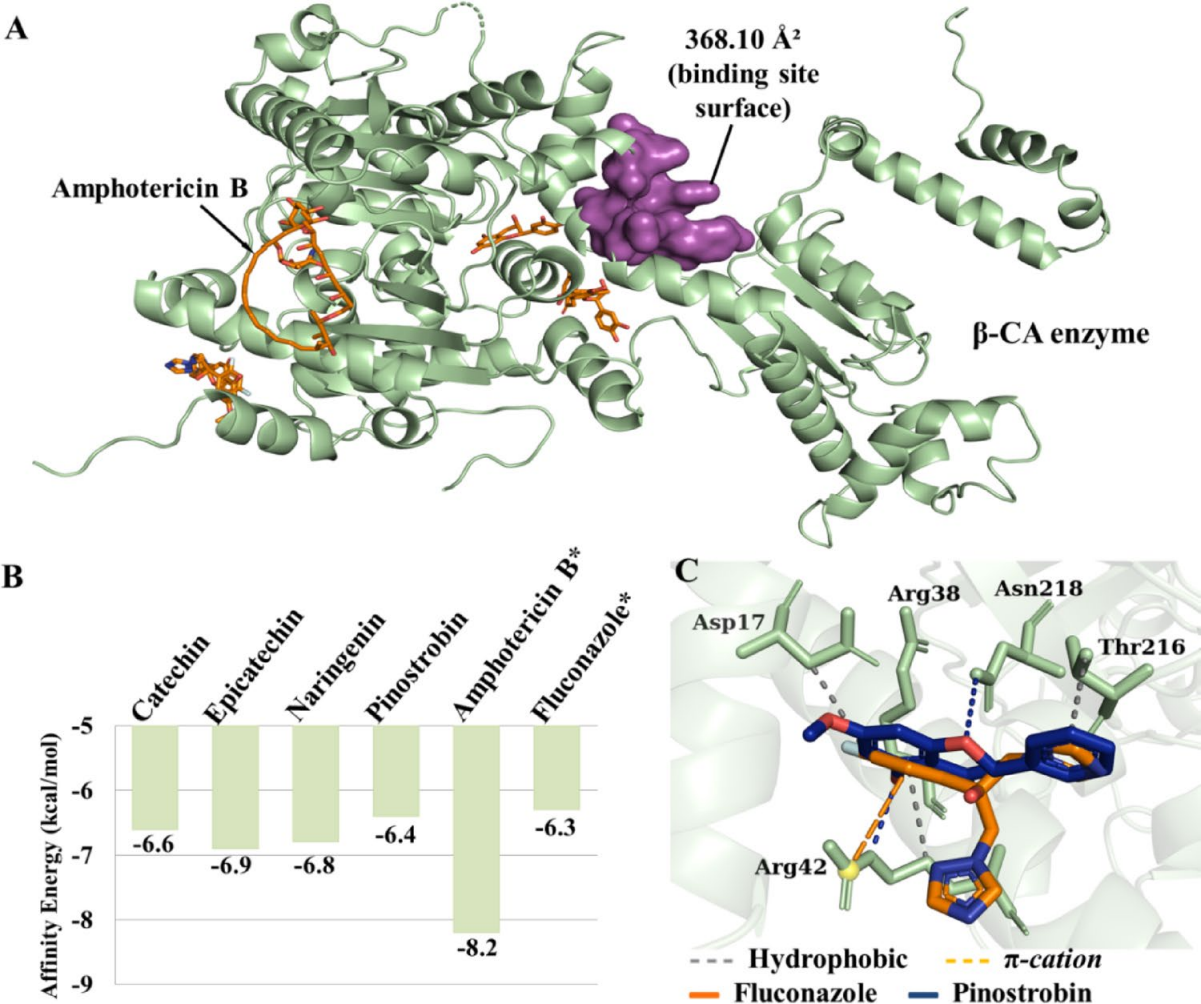
**A** Three-dimensional image of the β-CA protein and binding cavity, **B** affinity energy (kcal/mol) of the leads compounds and controls*, **C** binding site of fluconazole and pinostrobin

In *Cryptococcus neoformans*, β-CA is a critical enzyme that acts as a CO2 sensor and is essential for regulating intracellular pH. These processes are fundamental for the pathogen’s virulence and its ability to adapt to the host environment, making it a vital target for investigating the multi-target mechanism of action of cashew phytochemicals (Rathore et al. 2022; Zhao et al. 2023). The molecular docking results (Fig. 8a) demonstrated that catechin exhibited an *E*_A_ of −6.6 kcal/mol, while epicatechin demonstrated a value of −6.9 kcal/mol. Furthermore, naringenin and pinostrobin exhibited an *E*_A_ of −6.8 kcal/mol and − 6.4 kcal/mol, respectively. The results of the study demonstrated that Amphotericin B and fluconazole exhibited *E*_A_ of −8.2 kcal/mol and − 6.3 kcal/mol, respectively (see Fig. 8b). The predicted protein cavity has a molecular surface area of 368.10 Å^2^ (see Fig. 8a), and it constitutes a hydrophobic cavity due to the predominance of apolar and aromatic side chain residues. It has been predicted that the following amino acid residues will form a cavity: Gly200A, Phe202A, Pro137C, Leu138C, Pro139C, Pro144C, Gly145C, Gly146C, and Val150C. The aforementioned cavity also includes the side chains of the following amino acid residues: Glu201A, Arg203A, Gln136C, Asn143C, and Thr147C. The molecular docking results demonstrated that pinostrobin interacted with a residue in the binding site of the fluconazole control (Arg42C), suggesting a potential analogous effect to that of the antifungal agent employed (Fig. 8c). The data demonstrated that no interactions were observed between the other lead compounds and either the predicted cavity or the binding site of the amphotericin B control. Therefore, the administration of these compounds may not be sufficient to reverse the proliferation process of *C. neoformans* under environmental conditions.

Pinostrobin stood out in this target as the only lead compound to interact with the Arg42C residue within the binding site. Since this is the same residue targeted by the fluconazole control, it suggests a potential analogous mechanism of action to that of commercial antifungal agent for this specific compound. Therefore, the lack of interaction with the predicted cavity or the control binding site suggests that β-CA inhibition catechin, epicatechin, and naringenin, alone may not be sufficient to reverse fungal proliferation. Nevertheless, the overall potential of these leads is supported by an integrative approach. Their primary antifungal activity is likely driven by their higher specificity and stable interactions with the other two targets, CnFTase and AdSS.

**Fig. 9:**
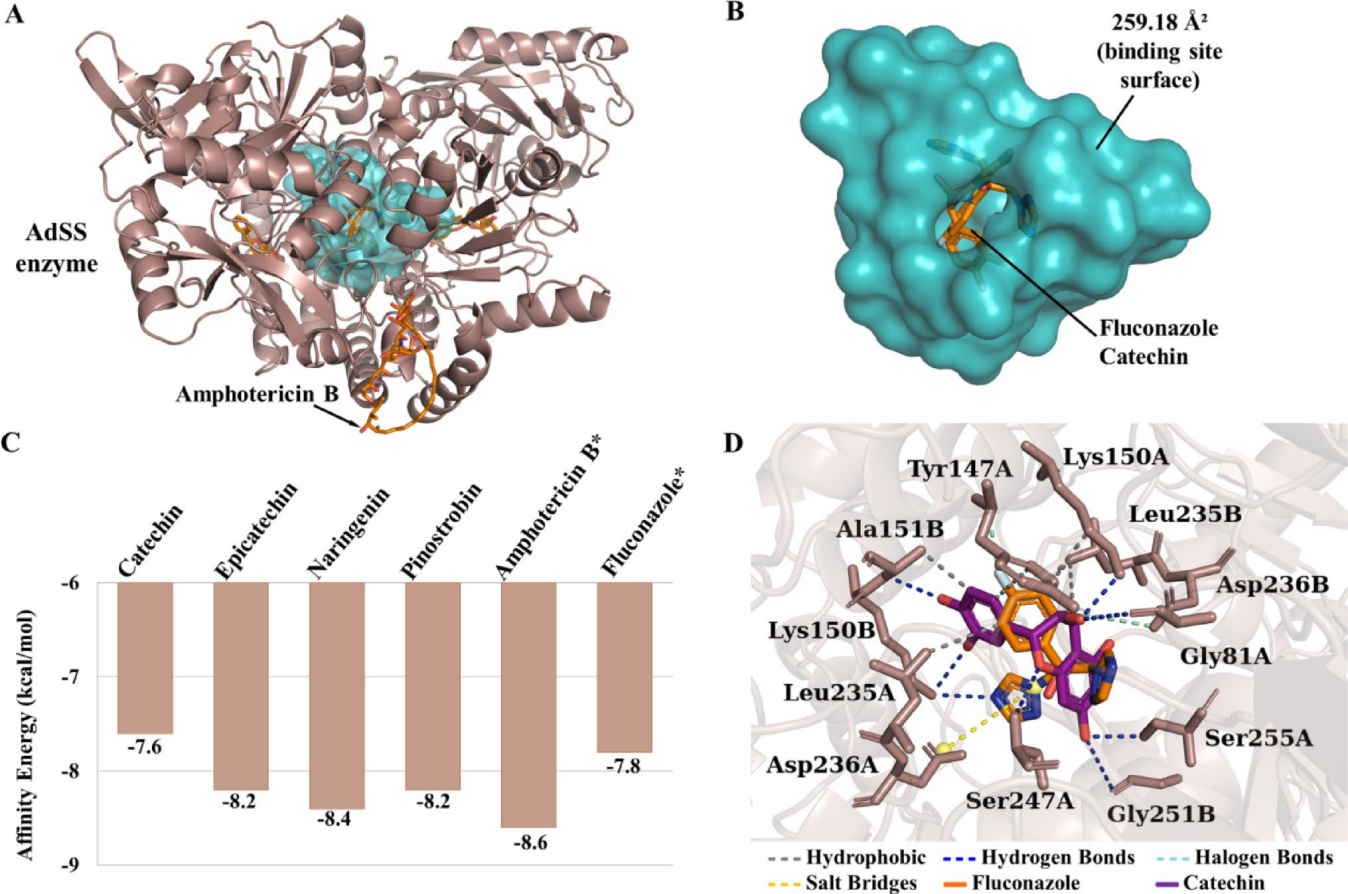
**A** Three-dimensional image of the AdSS protein, **B** the binding cavity, **C** affinity energy (kcal/mol) of the leads compounds and controls*, **D** binding site of fluconazole and catechin

In the context of AdSS, it was observed that the lead compounds exhibited favourable affinity energy, with the catechin compound demonstrating an affinity energy of −7.6 kcal/mol, epicatechin exhibiting a value of −8.2 kcal/mol, naringenin displaying an affinity energy of −8.4 kcal/mol, and pinostrobin showing a value of −8.2 kcal/mol. The affinity energies of Amphotericin B and fluconazole were found to be equal to −8.6 kcal/mol and − 7.8 kcal/mol, respectively (see Fig. 9c). The molecular docking results suggest that, among the lead compounds, catechin is complexed in the predicted cavity, where the fluconazole control is located, indicating that catechin may present inhibitory activity similar to the antifungal (Fig. 9b and d). The molecular surface of this cavity is 259.18 Å^2^ (see Fig. 9b), and it is located between chains A and B (see Fig. 9a). The amino acid residues Cys38, Ala39, Gly81, Tyr147, Lys150, Ala151, Leu235, Asp236, Ser245, Ser246, Ser247, Gly252, Ser255 and Gly256 are located on the surface. The other lead compounds are not inserted in the interaction cavity, demonstrating lower specificity for AdSS.

### Molecular dynamics simulations

**Fig. 10:**
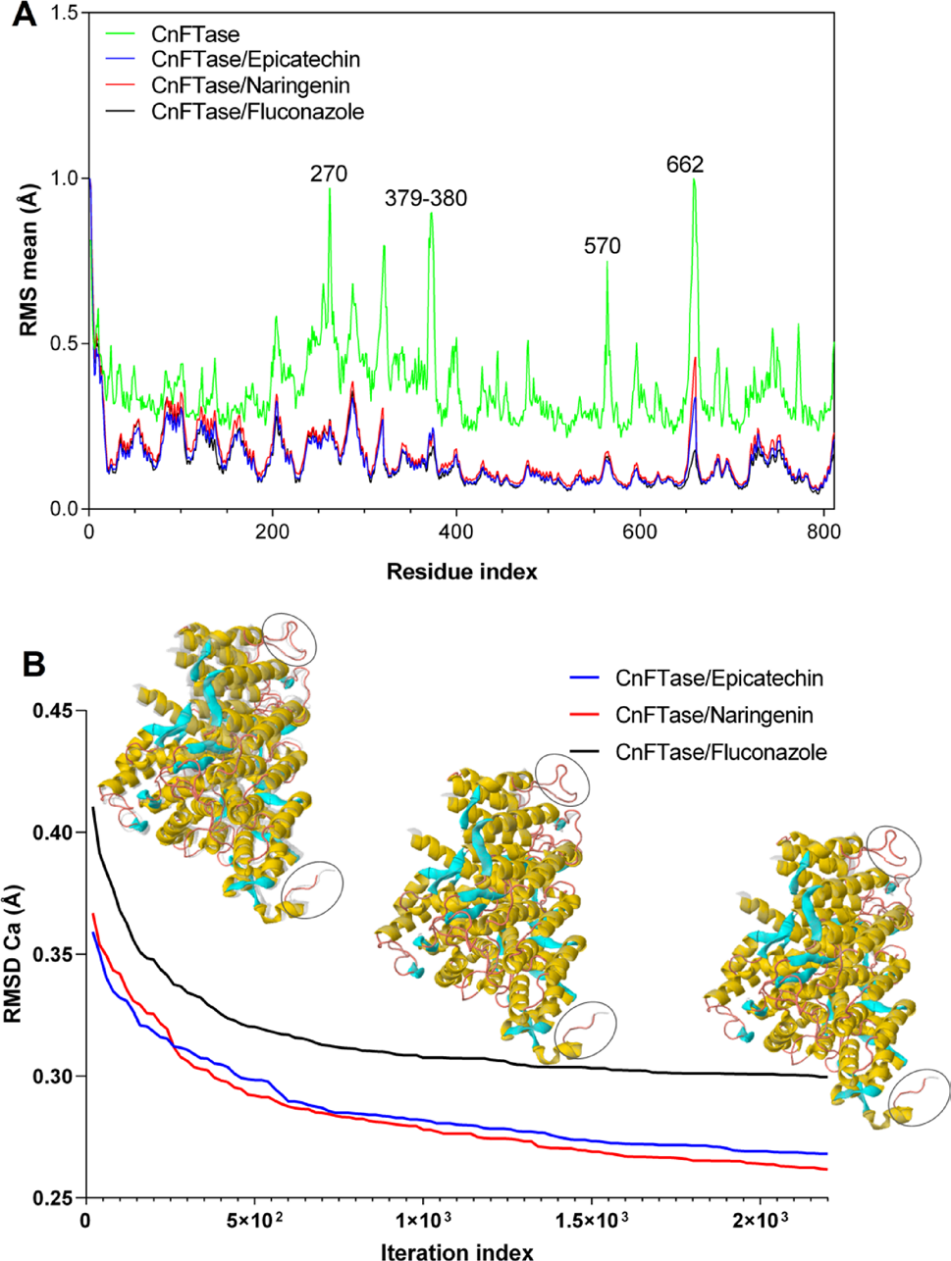
**A** Conformational variations analyzed in RMS mean (Å) in comparison with the complexes. **B** Convergence of the morphing trajectory for the complexes CnFTase-Fluconazole (black line), CnFTase-Epicatechin (blue line), and CnFTase-Naringenin (red line), indicating the RMSD variation of Ca (in Å) in relation to the iteration index. The highlighted region in a circle reveals the conformational variation observed after complexation of the ligands to the receptor

MD simulations were conducted using a normal mode analysis (NMA) module to observe the flexibility of the ligand-protein complex formed between the lead compounds (epicatechin and naringenin) and the receptor CnFTase. The results demonstrated that the NMA module applied to the crystallized structure of CnFTase (Fig. 10) generated an average RMS, observed to have peaks with a flexibility index of approximately 1.0 Å for residues 270, 379–380, 570, and 662. Whereas CnFTase-Fluconazole (black line), CnFTase-epicatechin (blue line), and CnFTase-naringenin (red line) produced a significantly low and an average RMS mean, indicating decreased flexibility of the residues in CnFTase after the formation of protein-ligand complexes with RMS mean values that are lower than 0.5 Å (Fig. 10A).

Upon analysis of the iteration trajectory, it was ascertained that the NMA mode indicated a smaller conformational torsion of the Cα of the CnFTase structure at the inception and conclusion of the MD simulations (Fig. 10B). In the trajectory of the CnFTase-Fluconazole complex (black line), it is possible to observe an initial RMSD of approximately 0.437 Å, with convergence iteration of approximately 2.200 × 10^3^ (calculation step), resulting in an last RMSD of 0.298 Å (Fig. 10B). The calculated value lower expresses a plausible physiological movement, without exaggeration or structural collapse (López-Blanco et al. 2014; Da Rocha et al. 2024). For the CnFTase-epicatechin complex (blue line), an initial RMSD of approximately 0.379 Å is observed, where the structure stabilizes with an RMSD of 0.263 Å and convergence iteration in the order of 2.200 × 10^3^ (Fig. 10B). For the CnFTase-naringenin (red line), an initial RMSD of approximately 0.380 Å is observed, where the structure stabilizes with an RMSD of 0.257 Å and convergence iteration in the order of 2.200 × 10^3^ (Fig. 10B). The results of the NMA-based MD simulations suggest that collective movements for both complexes are stable.

**Fig. 11:**
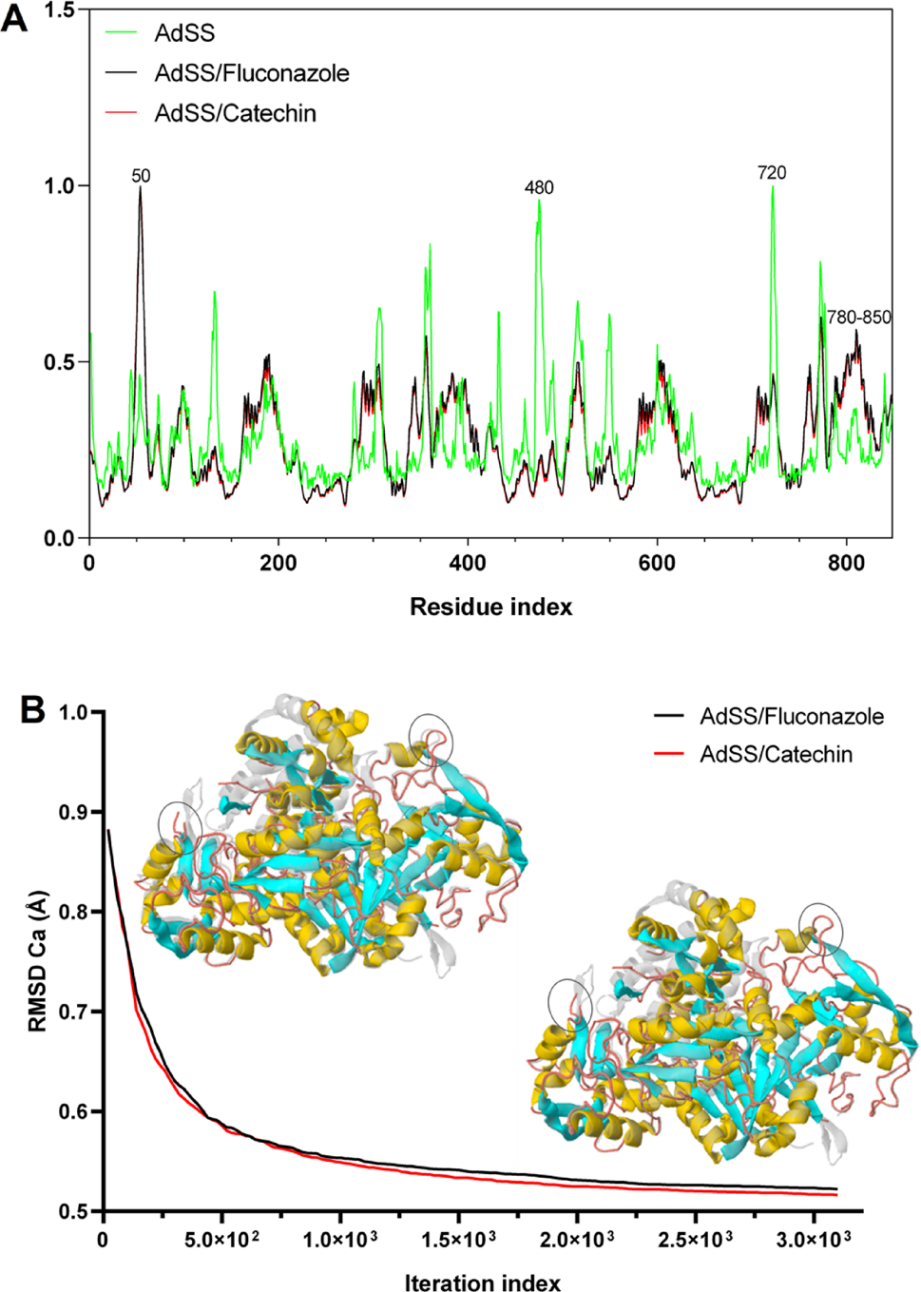
**A** Conformational variations analyzed in RMS mean (Å) in comparison with the complexes receptor-ligand. **B** Convergence of the morphing trajectory for the complexes AdSS-Fluconazole (black line), AdSS-Catechin (red line), indicating the RMSD variation of Ca (in Å) in relation to the iteration index. The highlighted region in a circle reveals the conformational variation observed after complexation of the ligands to the receptor

The flexibility analysis (Fig. 11A) of AdSS (green line) generated an average RMS, observed to have peaks with a flexibility index of approximately 1.0 Å for residues 480 and 720. Whereas AdSS-Fluconazole (black line) and AdSS-Catechin (red line) produced an average RMS lower, indicating decreased flexibility of the residues in AdSS after the formation of protein-ligand complexes, except residue 50 and residue sequence 780–850 (Fig. 11A).

Upon analysis of the iteration trajectory, Catechin exhibits similar action to Fluconazole against AdSS with an initial RMSD in the order of 0.920 Å. The final RMSD of AdSS-Fluconazole and AdSS-Catechin complexes decreased to a value of 0.519 Å and 0.514Å, respectively, with convergence iteration of approximately 3.100 × 10^3^ (calculation step), indicating a smaller conformational torsion of the Cα of the receptor structure (Fig. 11B). These results revealed similar variations in Cα RMSD for the AdSS-Fluconazole and AdSS-Catechin complexes. The results of the NMA-based MD simulations suggest that the collective movements of both complexes are stable.

## Conclusion

The integrative approach combining in silico pharmacokinetic and pharmacodynamic prediction techniques suggests that phytochemicals from A. occidentale represent promising lead compounds against *C. neoformans*. Among the analyzed molecules, catechin, epicatechin, naringenin, and pinostrobin were identified as lead compounds through multiparametric optimization. Molecular docking provided theoretical insights into their potential inhibitory action, showing high binding affinities for CnFTase and AdSS, frequently interacting with residues within the fluconazole binding site and predicted cavities. Furthermore, MD simulations demonstrated that epicatechin, naringenin (CnFTase), and catechin (AdSS) form stable complexes with structural flexibility comparable to fluconazole. Nevertheless, since these findings are based on purely computational models, in vitro and in vivo experimental studies are needs to validate the biological efficacy and safety of these cashew-derived flavonoids as novel antifungal agents.

## Supplementary Information

Below is the link to the electronic supplementary material.

Supplementary Material 1

## Data Availability

All the data generated or analyzed during this study are included in this published article.
