# Supplementary material for: Bioactive flavonoids from Anacardium occidentale as promising natural inhibitors of Cryptococcus neoformans: a computational perspective on secondary metabolites against critical fungal pathogens
Source: Arch Microbiol. 2026 Mar 3;208(5):234. doi: 10.1007/s00203-026-04814-9 (PMC12957122; doi:10.1007/s00203-026-04814-9)
Supplement: Supplementary file 1 — Supplementary Material 1 [file 203_2026_4814_MOESM1_ESM.docx]

**Bioactive Flavonoids from *Anacardium occidentale* as Promising Natural Inhibitors of *Cryptococcus neoformans*: A Computational Perspective on Secondary Metabolites against Critical Fungal Pathogens**

**Marcus Vinícius Ferreira da Silva^1^, Jacilene Silva^2^, Victor Moreira de Oliveira^3^ Matheus Nunes da Rocha^4^, Selene Maia de Morais^1^, Emmanuel Silva Marinho^1,4*^**

^1^ Postgraduate Program in Veterinary Sciences, State University of Ceará, Fortaleza, Ceará, Brazil.

^2^ Department of Biological Chemistry, Regional University of Cariri, Crato, Ceará, Brazil.

^3^ Postgraduate Program in Biotechnology, State University of Ceará, Fortaleza, Ceará, Brazil.

^4^ Postgraduate Program in Natural Sciences, State University of Ceará, Fortaleza, Brazil.

* Correspondence: Science and Technology Center – Chemistry Course, State University of Ceará, Fortaleza, Brazil. E-mail address: emmanuel.marinho@uece.br

--Supplementary data--

**Table S1. RMSD and types of interactions formed between ligands and the CnFTase.**

| **Ligand** | **RMSD (Å)** | **Affinity Energy (kcal/mol)** | **Interaction Type** | **Residue (Distance in Å)** |
| --- | --- | --- | --- | --- |
| **Apigenin** | 1.725 | -9.8 | Hydrophobic | Tyr109A (3.67), Leu116A (3.43), Ala149A (3.69), Trp153A (3.98), Val194B (3.84) |
|  |  |  | H-Bond | His113A (1.81), His146A (2.41), Arg181B (2.85), Ser238B (2.18), Tyr269B (3.10) |
| **Catechin** | 1.685 | -8.8 | Hydrophobic | Trp112A (3.51), Ala149A (3.65), Trp153A (3.75), Glu193B (3.56), Ala237B (3.85) |
|  |  |  | H-Bond | His113A (2.43), Arg181B (3.07), Arg181B (2.34), Asp195B (2.36) |
| **Epicatechin** | 1.399 | -9.4 | Hydrophobic | His146A (3.67), Val194B (3.85), Ala237B (3.60) |
|  |  |  | H-Bond | His146A (2.27), His146A (2.64), Tyr150A (3.60), Arg181B (2.16), Arg181B (2.09), Val194B (2.32), Ser238B (2.69) |
| **Galangin** | 1.307 | -9.2 | Hydrophobic | Tyr109A (3.67), Leu116A (3.90), Ala149A (3.74), Trp153A (3.85), Asp195B (3.82), Phe239B (3.90) |
|  |  |  | H-Bond | His146A (2.37), Ala149A (2.71), Asp195B (2.37) |
|  |  |  | π-Stacking | Trp153A (4.53) |
| **Kaempferol** | 1.810 | -9.3 | Hydrophobic | Tyr109A (3.76), Ala149A (3.58), Trp153A (4.00), Val194B (3.83), Ala237B (3.89) |
|  |  |  | H-Bond | His113A (1.82), His146A (2.49), Arg181B (3.02), Tyr269B (3.16) |
| **Naringenin** | 1.393 | -9.3 | Hydrophobic | His146A (3.71), Val194B (3.79), Ala237B (3.85) |
|  |  |  | H-Bond | His146A (2.23), His146A (2.65), Arg181B (2.18), Arg181B (2.32), Asp195B (2.60), Ser238B (3.47) |
|  |  |  | π-Stacking | Trp153A (5.50) |
| **Pinostrobin** | 1.983 | -8.8 | Hydrophobic | Tyr109A (3.59), Leu116A (3.30), Val194B (3.83), Ala237B (3.84), Phe239B (3.70) |
|  |  |  | H-Bond | His113A (1.79) |
| **Rutin** | 0.851 | -9.7 | Hydrophobic | Gln228B (3.99) |
|  |  |  | H-Bond | Tyr230B (2.78), Lys239A (2.78), His240A (2.14), Arg259B (2.34), Ser261B (2.95), Ser291B (2.41), Ser291B (3.15) |
| **Mono-unsaturated AA** | 1.263 | --7.2 | Hydrophobic | Trp90B (3.75), Trp90B (3.87), Tyr109A (3.84), Tyr409B (3.26), Tyr409B (3.71) |
|  |  |  | H-Bond | Ser87B (3.60), Tyr109A (2.70), Gln110A (2.24), Gln110A (2.36), Arg197B (2.79) |
|  |  |  | π-Stacking | Tyr109A (5.00) |
| **Di-unsaturated AA** | 1.626 | -7.2 | Hydrophobic | Leu84B (3.36), Trp90B (3.91), Trp90B (3.46), Trp94B (3.48), Trp329B (3.95), Tyr409B (3.34) |
|  |  |  | H-Bond | Tyr409B (2.48) |
|  |  |  | π-Stacking | Tyr409B (3.84) |
| **Tri-unsaturated AA** | 1.911 | -7.8 | Hydrophobic | Trp90B (3.58), Trp90B (3.78), Trp90B (3.83), Trp94B (3.74), Trp329B (3.63), Tyr409B (3.65) |
|  |  |  | H-Bond | Tyr409B (2.79) |
| **Di-unsaturated Cardol** | 1.331 | -7.3 | Hydrophobic | Trp90B (3.70), Trp90B (3.59), Trp94B (3.57), Trp94B (3.66), Tyr409B (3.78), Tyr409B (3.39) |
|  |  |  | H-Bond | Tyr409B (2.63), Tyr409B (2.25) |
| **Tri-unsaturated Cardol** | 1.692 | -7.1 | Hydrophobic | Leu84B (3.86), Trp94B (3.97), Trp94B (3.41), Tyr109A (3.71), Tyr409B (3.97), Tyr409B (3.70) |
|  |  |  | H-Bond | Trp94B (2.87) |
|  |  |  | π-Stacking | Trp90B (5.18), Trp90B (4.83) |
| **Amphotericin B*** | 1.179 | -10.3 | Hydrophobic | Ala72A (3.72), Trp94B (3.22), Tyr109A (3.75), Arg405B (3.82), Tyr409B (3.50) |
|  |  |  | H-Bond | Ser83B (3.29), Ser87B (2.27), Lys107A (3.02), Asp407B (2.47), Asp407B (1.88), Asp407B (2.13) |
|  |  |  | Salt Bridges | Lys80B (4.58) |
| **Fluconazole*** | 1.518 | -8.2 | Hydrophobic | Val194B (3.74) |
|  |  |  | H-Bond | His113A (2.42), Val194B (2.36), Val194B (2.98) |
|  |  |  | π-Stacking | Trp153A (5.50) |
|  |  |  | Salt Bridges | Asp195B (5.18) |

**Legend: AA (anacardic acid); *Controls.**

**Table S2. RMSD and types of interactions formed between ligands and the β-CA.**

| **Ligand** | **RMSD (Å)** | **Affinity Energy (kcal/mol)** | **Interaction Type** | **Residue (Distance in Å)** |
| --- | --- | --- | --- | --- |
| **Apigenin** | 1.344 | -7.1 | Hydrophobic | Gln104B (3.25), Gln104B (3.92), Pro148C (3.68), Pro188B (3.70) |
|  |  |  | H-Bond | Thr189B (3.31), His229A (2.52) |
| **Catechin** | 1.407 | -6.6 | Hydrophobic | Gln104B (3.54), Pro188B (3.47) |
|  |  |  | H-Bond | Gln104B (2.41), Arg151C (2.02), Thr189B (2.11), Glu204A (1.97), His229A (3.31), His229A (2.23), Phe231A (3.32), Phe231A (3.57) |
| **Epicatechin** | 1.359 | -6.9 | H-Bond | Lys97C (2.19), Glu99C (2.43), Asp101C (2.20), Asp101C (3.21), Ser102C (2.69), Arg151C (2.60) |
| **Galangin** | 1.834 | -6.6 | Hydrophobic | Glu13C (3.67), Asp17C (3.65), Thr45C (3.58), Thr216B (3.93) |
|  |  |  | H-Bond | Asp17C(2.53), Arg38C (3.57), Arg42C (3.06), Thr216B (2.91), Asn218B (3.28) |
|  |  |  | π-Cation | Arg42C (4.39) |
| **Kaempferol** | 1.825 | -6.9 | Hydrophobic | Asp101B (3.68) |
|  |  |  | H-Bond | Lys97B (2.15), Lys97C (2.13), Ser102C (2.67), Ser102C (2.42), Ser102C (3.24) |
| **Naringenin** | 1.348 | -6.8 | Hydrophobic | Gln104B (3.12), Asn108B (3.93), Pro148C (3.54), Pro188B (3.89) |
|  |  |  | H-Bond | Pro98B (2.59), Glu99B (3.65), Arg151C (3.37), His229A (2.30) |
| **Pinostrobin** | 1.427 | -6.4 | Hydrophobic | Asp17C (3.98), Thr216B (3.71) |
|  |  |  | H-Bond | Arg42C (2.24), Asn218B (2.97) |
| **Rutin** | 1.951 | -8.5 | H-Bond | Asn62A (2.14), Asn62A (2.37), Gln136C (2.37), Gly145C (2.33), Gly146C (2.48), Arg203A (2.39) |
| **Mono-unsaturated AA** | 1.643 | -4.7 | Hydrophobic | Val76B (3.61), Thr77B (3.76), Ala80B (3.96) |
|  |  |  | H-Bond | Thr77C (2.08) |
| **Di-unsaturated AA** | 1.886 | -4.4 | Hydrophobic | Glu99B (3.76), Glu99C (3.96) |
|  |  |  | H-Bond | Asp101C (2.30), Ser102C (2.28) |
| **Tri-unsaturated AA** | 1.441 | -4.3 | Hydrophobic | Phe51A (3.58), Phe51A (3.51), Glu54A (3.45), Lys82A (3.65) |
| **Di-unsaturated Cardol** | 1.698 | -3.9 | Hydrophobic | Phe51A (3.65), Phe51A (3.35), Glu54A (3.69), Gln55A (3.88) |
| **Tri-unsaturated Cardol** | 1.786 | -3.9 | Hydrophobic | Glu48A (3.65), Phe51A (3.39), Glu54A (3.90), Glu54A (3.98), Gln55A (3.61), Lys82A (3.76) |
|  |  |  | H-Bond | Glu50A (3.23) |
| **Amphotericin B*** | 1.415 | -8.2 | Hydrophobic | Glu35C (3.86), Tyr39C (3.57) |
|  |  |  | H-Bond | Glu32C (3.17), Trp40B (2.00), Glu48B (2.90), Val76B (2.77) |
|  |  |  | Salt Bridges | Arg81B (5.04) |
| **Fluconazole*** | 1.030 | -6.3 | Hydrophobic | Arg38C (3.59), Arg42C (3.75) |
|  |  |  | π-Cation | Arg42C (4.31) |

**Legend: AA (anacardic acid); *Controls.**

**Table S3. RMSD and types of interactions formed between ligands and the AdSS.**

| **Ligand** | **RMSD (Å)** | **Affinity Energy (kcal/mol)** | **Interaction Type** | **Residue (Distance in Å)** |
| --- | --- | --- | --- | --- |
| **Apigenin** | 1.208 | -8.7 | Hydrophobic | Asn43A (3.72) |
|  |  |  | H-Bond | Asp18A (3.37), Asn43A (3.19), Thr139A (3.30), Arg153B (3.72), Thr244A (2.78) |
| **Catechin** | 1.888 | -7.6 | Hydrophobic | Tyr147A (3.99), Ala151B (3.14), Leu235B (3.95) |
|  |  |  | H-Bond | Gly81A (3.71), Lys150A (1.95), Lys150B (2.57), Ala151B (2.81), Ser247A (3.07), Gly251B (3.30), Ser255A (2.13) |
| **Epicatechin** | 1.504 | -8.2 | Hydrophobic | Leu233B (3.54), Leu233B (3.70) |
|  |  |  | H-Bond | Trp16B (2.76), Asp18B (2.81), Val278B (1.92) |
| **Galangin** | 1.694 | -8.5 | Hydrophobic | Asn43B (3.57), Thr139B (3.83) |
|  |  |  | H-Bond | Thr244B (2.30) |
| **Kaempferol** | 1.194 | -9.0 | Hydrophobic | Asn43B (3.60), Thr139B (3.87) |
|  |  |  | H-Bond | Trp16B (3.25), Asp18B (2.79), Asp18B (2.72), Thr244B (2.30) |
| **Naringenin** | 1.639 | -8.4 | Hydrophobic | Asn43B (3.64), Thr139B (3.91), Leu233B (3.46) |
|  |  |  | H-Bond | Asp18B (2.81), Ala44B (2.19), Arg153A (3.34), Asn229B (2.73), Thr244B (2.29) |
| **Pinostrobin** | 1.854 | -8.2 | Hydrophobic | Asn43A (3.82), Thr139A (3.94) |
|  |  |  | H-Bond | Asn229A (2.67), Thr244A (2.36), Arg308A (3.46), Arg308A (2.28) |
| **Rutin** | 0.743 | -9.4 | Hydrophobic | Thr304A (3.88) |
|  |  |  | H-Bond | Asn43A (3.28), Gly45A (3.55), Asn63A (2.51), Lys141A (3.50), Asn229A (3.17), Asn229A (3.21), Thr304A (2.05), Thr304A (2.24), Thr304A (2.83), Val305A (2.40), Arg308A (2.46), Arg308A(3.41), Arg310A (2.35), Arg310A (2.30) |
|  |  |  | Salt Bridges | Arg308A (5.11), Arg310A (5.46) |
| **Mono-unsaturated AA** | 0.863 | -7.6 | Hydrophobic | Tyr147A (3.71), Tyr147A (3.37), Tyr147B (3.46), Tyr147B (3.76), Ala151A (3.21), Ala151B (3.36), Leu235A (3.62), Leu235B (3.67) |
|  |  |  | H-Bond | Ser247B (2.45), Ser255B (2.82) |
|  |  |  | Salt Bridges | Lys150B (4.27) |
| **Di-unsaturated AA** | 1.676 | -8.1 | Hydrophobic | Ala39B (3.71), Tyr147A (3.64), Tyr147B (3.77), Tyr147B (3.55), Lys150B (3.96), Ala151A (3.77), Ala151B (3.83), Leu235A (3.96), Leu235B (3.77) |
|  |  |  | H-Bond | Asp236B (2.68), Ser247B (2.63), Ser255A (2.38) |
|  |  |  | Salt Bridges | Lys150A (5.47) |
| **Tri-unsaturated AA** | 0.941 | -8.1 | Hydrophobic | Tyr147A (3.48), Tyr147A (3.25), Tyr147B (3.56), Lys150A (3.82), Lys150B (3.52), Ala151A (3.47), Ala151B (3.54), Leu235B (3.68) |
|  |  |  | H-Bond | Ser247B (2.12) |
|  |  |  | Salt Bridges | Lys150B (4.58) |
| **Di-unsaturated Cardol** | 1.146 | -8.2 | Hydrophobic | Ala39A (3.89), Tyr147A (3.67), Tyr147A (3.77), Tyr147B (3.61), Ala151A (3.39), Leu235A (3.23), Leu235B (3.58) |
|  |  |  | H-Bond | Ala151A (3.04) Ala151B (3.26) |
| **Tri-unsaturated Cardol** | 1.550 | -7.8 | Hydrophobic | Ala39A (3.83), Tyr147A (3.58), Tyr147A (3.57), Tyr147B (3.46), Lys150A (3.63), Lys150B (3.99), Ala151B (3.65), Leu235A (3.44), Leu235B (3.46) |
|  |  |  | H-Bond | Asp236A (2.16), Ser247B (2.35), Ser247B (3.30) |
| **Amphotericin B*** | 1.830 | -8.6 | Hydrophobic | Ile354A (3.78), Arg368A (3.63) |
|  |  |  | H-Bond | Asp111B (2.86), Asp111B (2.40), Phe162B (2.66), Phe162B (2.66), Asp355A (2.89), Glu360A (3.16), Glu360A (3.56), Arg368A (3.34), Arg368A (2.71) |
|  |  |  | Salt Bridges | Arg368A (4.91), Lys371A (4.26) |
| **Fluconazole*** | 1.878 | -7.8 | Hydrophobic | Lys150A (3.88), Leu235A (3.83), Leu235B (3.63) |
|  |  |  | H-Bond | Lys150B (2.59) |
|  |  |  | Halogen Bonds | Tyr147A (3.15), Asp236B (3.62) |
|  |  |  | Salt Bridges | Asp236A (5.48) |

**Legend: AA (anacardic acid); *Controls.**
